# Effectiveness of betahistine (48 mg/day) in patients with vestibular vertigo during routine practice: The VIRTUOSO study

**DOI:** 10.1371/journal.pone.0174114

**Published:** 2017-03-30

**Authors:** Vladimir A. Parfenov, Volodymyr A. Golyk, Eduard I. Matsnev, Svetlana V. Morozova, Oleg A. Melnikov, Ludmila M. Antonenko, Elena E. Sigaleva, Maksym I. Situkho, Olena I. Asaulenko, Vasyl I. Popovych, Maxim V. Zamergrad

**Affiliations:** 1 Neurology Department, I.M. Sechenov First Moscow State Medical University, Moscow, Russia; 2 Neurology and Border States Department, Ukrainian State Institute of Medical and Social Problems of Disability, Ministry of Public Health of Ukraine, Dnipropetrovsk, Ukraine; 3 State Scientific Centre of the Russian Federation, Institute of Biomedical Problems of Russian Academy of Sciences, Moscow, Russia; 4 ENT Department, I.M. Sechenov First Moscow State Medical University, Moscow, Russia; 5 ANO Guta Clinic, Moscow, Russia; 6 Clinical Audiology and Vestibology Department, O.S. Kolomyichenko Otolaryngology Institute, Kyiv, Ukraine; 7 Kyiv City Clinical Hospital № 9, Neurology Department, Kyiv, Ukraine; 8 P.L. Shupyk National Medical Academy of Postgraduate Education, Kyiv, Ukraine; 9 ENT Department, Ivano-Frankivsk Regional Clinical Hospital, Ivano-Frankivsk, Ukraine; 10 Ivano-Frankivsk National Medical University, Ivano-Frankivsk, Ukraine; 11 Department of Neurology, Russian Medical Academy of Postgraduate Education, Moscow, Russia; Cardiff University, UNITED KINGDOM

## Abstract

**Background:**

Vestibular vertigo is associated with substantially reduced quality of life. Betahistine is effective in improving vertigo-associated symptoms, with longer treatment periods leading to greater improvements; however, it is not known whether these effects persist after treatment cessation.

**Methods:**

VIRTUOSO was a prospective, multinational, non-comparative, post-marketing observational programme investigating the effectiveness of betahistine (48 mg/day) and the course of vertigo after the discontinuation of treatment. Patients with vestibular vertigo who were prescribed 48 mg/day betahistine were enrolled in Russia and Ukraine. Treatment duration was up to 2 months, and patients were followed up for 2 months after discontinuation of betahistine. Efficacy endpoints included clinical response (assessed by change in vertigo severity), monthly attack frequency, and physician and patient grading of overall clinical response and improvement of vertigo-associated symptoms.

**Results:**

Overall, 309 patients were enrolled and 305 completed the study. Clinical response was rated as good, very good or excellent in 74.1% of patients at end of treatment, with vertigo severity significantly decreased from baseline (*p* < 0.001). Monthly vertigo attack frequency decreased significantly during the 2 months of treatment (*p* < 0.001 from baseline) and further decreased during the 2-month follow-up (*p* < 0.001 from end of treatment). Overall, clinical response was graded as good or excellent by 94.4% of physicians and 95.4% of patients. Clinical improvement was considered either good or excellent by 82.6–90.5% of physicians and patients for nausea, vomiting and faintness. Only one adverse event was reported, with no serious adverse events.

**Conclusion:**

Our findings suggest that betahistine (48 mg/day) therapy is effective in treating vertigo in routine clinical settings. The observed effects persisted for 2 months after treatment cessation, suggesting that betahistine may facilitate lasting vestibular compensation.

## Introduction

Vertigo and dizziness are among the most frequent symptoms in medical practice, with a lifetime prevalence of 17.0–30.0% [1] and annual prevalence of 16.7–27.0% [2,3] reported in the general population. In a study of 2064 working-age people in the community, results from a survey showed that over 20% (*n* = 480) of respondents had suffered from dizziness in the previous month and 30% of these respondents had dizziness that lasted more than 5 years [4]. Vertigo and dizziness are associated with a lower health-related quality of life and a negative impact upon daily living [2,5].

Vestibular vertigo is characterised by illusion of movement and spatial orientation [5]. It can be further classified as central or peripheral, depending on whether the vertigo is caused by lesions of the central or peripheral parts of the vestibular system [6]. Vestibular vertigo affects 1.8–4.9% of adults every year [3,5], is estimated to affect 3.0–10.0% of adults in their lifetime [1] and is associated with a significantly (*p* ≤ 0.001) higher occurrence of medical consultation, sick leave, interruption of daily activities, and avoidance of leaving the house compared with non-vestibular dizziness [2]. Risk factors associated with vestibular vertigo include female sex, depression, hypertension and dyslipidaemia [5]. Peripheral vestibular disorders causing vertigo include Ménière’s disease, benign paroxysmal positional vertigo and vestibular neuronitis [6].

Medical treatments administered for vestibular vertigo vary depending on aetiology; for Ménière’s disease, medical treatment options include salt restriction, diuretics, betahistine, and intratympanic injection of corticosteroids or gentamicin [6,7]. Betahistine is approved in >115 countries for the treatment of Ménière’s disease and the symptoms of vertigo. It is a structural analogue of histamine, and a weak agonist for histamine H(1) receptors and an antagonist for H(3) receptors [8]. It has been found to improve vestibular compensation in animal models of unilateral vestibular dysfunction, by increasing vestibulocochlear blood flow and reducing the histamine-induced excitatory response in vestibular cells by blocking local H(3) autoreceptors [8]. Several clinical trials have demonstrated that betahistine is effective in reducing the frequency and severity of vertigo, and improving vertigo-associated symptoms, including nausea and vomiting [7,9–15]. A 2016 Cochrane Review of randomised controlled trials of betahistine versus placebo in patients with symptoms of vertigo suggested that betahistine may have a positive effect in terms of reduction in vertigo symptoms; however, it was noted that the quality of available evidence is low [16]. Continued improvements in vertigo have been observed throughout betahistine treatment, and at a range of doses, for periods lasting from 45 days up to 12 months [7,11–15]; therefore, a longer duration of betahistine treatment may be required for the maximal effect of betahistine to be observed. Patient and investigator opinions of betahistine treatment have been high in these studies [9,10,12,13,17], suggesting that the effects of betahistine on vertigo symptoms may translate to reductions in the disease burden of vertigo. While betahistine has been a key part of the armamentarium for treating vertigo in general clinical practice for many years, the results of the recent Cochrane review highlight that more evidence is required to understand the size of any treatment benefit in terms of its ability to facilitate vestibular compensation [16]. Moreover, a greater understanding is needed of the extent to which this effect is maintained after treatment cessation.

The VIRTUOSO study was a multicentre, post-marketing observational programme in Russia and Ukraine that investigated the effectiveness of betahistine dihydrochloride (Betaserc^®^; Abbott Laboratories) when administered at the maximal recommended dose of 48 mg/day. It also aimed to assess the course of vestibular vertigo after discontinuation of betahistine treatment in routine clinical settings.

## Methods

### Study design

A prospective, multinational, non-comparative, post-marketing observational programme was conducted in 14 centres in the Russian Federation and 9 centres in Ukraine. The VIRTUOSO study (NCT01759251) [18] enrolled patients with vestibular vertigo who had been prescribed betahistine at the maximal recommended daily dose of 48 mg in accordance with the locally approved label. Physicians prescribed betahistine medication to the patients in routine outpatient clinical settings, and instructed them to take it orally at home, dividing the daily dose of 48 mg evenly throughout the day (two 8 mg tablets three times per day, one 16 mg tablet three times per day or one 24 mg tablet two times per day), preferably after meals. Treatment duration was 1–2 months, and patients were observed for approximately 2 months after discontinuation of betahistine. The following patient visits were scheduled: (1) baseline; (2) up to 30 days after first treatment dose of betahistine; (3) up to 60 days after first treatment dose of betahistine (end of treatment period); (4) 30 days from the last treatment dose of betahistine; (5) 60 days from the last treatment dose of betahistine (end of follow-up period). The treatment period lasted up to 60 days (from Visit 1 to Visit 3), and the follow-up period lasted for up to 60 days after the cessation of betahistine treatment (from Visit 3 to Visit 5).

### Patient recruitment and screening

Adult patients who could be treated with betahistine as per the locally approved label were recruited from routine outpatient clinical settings in Russia and Ukraine. Inclusion criteria for VIRTUOSO: patients ≥18 years of age with vestibular vertigo who had been prescribed 48 mg/day of betahistine in accordance with the locally approved label and started therapy ≤5 days prior to giving written consent. Main exclusion criteria: contraindications of betahistine treatment, middle- or inner-ear infection, psychiatric or significant neurological disorders, spinal-cord damage, use of any other agents for peripheral vestibular vertigo, ear surgery for vestibular disorders, pregnancy or breastfeeding, and previous betahistine therapy ≤4 weeks before start of treatment course.

VIRTUOSO was undertaken in accordance with Good Clinical Practice Guidelines and all relevant national guidelines, and patients provided written consent before enrolment. Approval for the study protocols were gained from the following regulatory authorities and independent ethics committees before patient enrolment: the Inter-Institutional Review Board in Russia and the Local Ethics Committees of Kyiv City Clinical Hospital № 9, Zaporizhzhia Medical Academy of Postgraduate Education, Crimean State National Medical University named after S.I. Georgievsky, Mykolaiv Clinical Hospital № 4, 30th City Otolaryngology Clinical Hospital, Sevastopol State Administration of MoH of Ukraine, Ivano-Frankivsk Regional Clinical Hospital, 3rd City Clinical Hospital and Institute of Urgent and Recovery Surgery named after V.K. Gusak NAMS of Ukraine.

### Efficacy variables

The primary objective of VIRTUOSO was to assess the effectiveness of betahistine (48 mg/day) treatment for vestibular vertigo in routine outpatient clinical settings, by assessing clinical response based on the Scale for Vestibular Vertigo Severity Level and Clinical Response Evaluation (SVVSLCRE) (S1 Fig). Secondary objectives were to assess improvement in vertigo and its associated symptoms over the treatment period, and to assess the course of vestibular vertigo during the follow-up period. Exploratory objectives were to describe the underlying diagnoses that account for vestibular vertigo according to ICD-10 (International Classification of Diseases 10th Revision) [19] and assess the course of vestibular vertigo during follow-up for subgroups of patients with different diagnoses.

Clinical response was assessed using the change in vestibular vertigo severity from baseline to end of treatment period. Symptom severity was graded on the SVVSLCRE (Level I [0–2] = absent vestibular vertigo; Level V [8–10] = very severe vestibular vertigo). Clinical response was described as worsening, no change, or by using a 4-point scale from moderate (reduced by 1 level) to excellent (reduced by 4 levels). Changes in SVVSLCRE level and monthly vertigo attack frequency were assessed from baseline to Visit 2 and the end of treatment period. The course of vestibular vertigo during the follow-up period was assessed using the change in monthly vertigo attack frequency from the end of treatment period to the end of first and second months of follow-up. Overall clinical response and improvement of vertigo-associated symptoms (tinnitus, hearing loss, nausea, vomiting, faintness and headache) were evaluated by physicians and patients on a 4-point scale from poor to excellent at Visit 2 and end of treatment. Patients were categorised according to ICD-10 in exploratory subgroup analyses, as described in the International Classification of Diseases 10th Revision [19] as this is the standard diagnosis tool for epidemiology, health management and clinical purposes [20].

### Safety variables

Vital signs and the incidence of adverse events (AEs) leading to discontinuation of betahistine therapy and serious adverse events (SAEs) were recorded.

### Sample-size determination

For VIRTUOSO, it was assumed that clinical response to betahistine would be achieved in at least 80% of the patients. For a sample size of 246 (without consideration of drop-out), a normal approximation two-sided 95% confidence interval (CI) for a single proportion would extend 5% (i.e. half the width of the CIs) from the observed proportion for an expected proportion of 80%. Assuming a 20% drop-out rate it was determined that the study population should constitute 308 patients. Therefore, the planned sample size for enrolment was 310 patients.

### Statistical analysis

Statistical analyses were conducted using SAS^®^ Version 9.2 (SAS, Cary, USA) under Windows^®^ 2008 Terminal (Microsoft, Redmond, USA). For the primary efficacy endpoint, the number and percentage of patients with each clinical response and the normal approximation two-sided marginal 95% CI for binominal proportions were presented. Statistical analyses were performed at α = 0.05 two-sided significance level; α between 0.05 and 0.1 was considered a trend toward significance. For the secondary endpoints, descriptive statistics including *p*-values and CIs were presented (comparisons between visits were performed using paired t-tests or Wilcoxon signed-rank tests).

### Changes to the study protocol

One protocol amendment was incorporated before any patients were included in the observational programme, which involved clarifying a figure and table, and making minor textual changes. These amendments did not affect the conduct or analyses of the study.

## Results

### Study population and disposition

**Fig 1:**
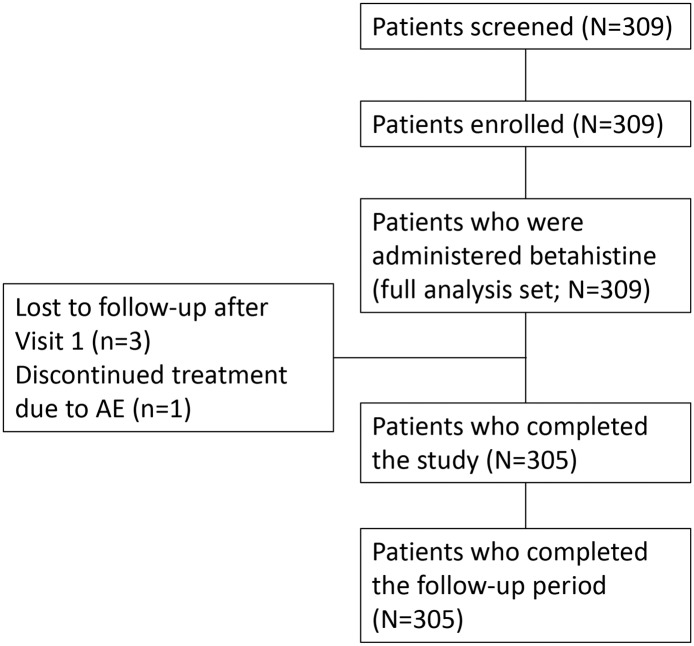
Flowchart of patient disposition.

Patient recruitment for VIRTUOSO took place from 15 January 2013 to 31 January 2014; the final patient follow-up visit was completed on 30 May 2014. A total of 309 patients were screened, and all 309 patients were enrolled and included in the full analysis set; efficacy analyses were undertaken for the 305 patients who completed the study and follow-up (60-day treatment group) (Fig 1). Out of the four patients that did not complete the study, three were lost to follow-up after Visit 1 and one discontinued treatment due to an AE. One additional patient had a combined Visit 2 and 3, which was only inputted as Visit 3.

Three protocol deviations were identified after study completion; two patients were prescribed a lower dose of betahistine (32 mg/day) until Visit 2 and one patient was administered betahistine for 86 days, instead of the 60-day treatment period specified in the protocol. All patients who had undergone protocol deviations were included in the efficacy analyses.

Patient demographics for the full analysis set are shown in Table 1. The most frequent diagnoses at enrolment, according to ICD-10 class, were diseases of vestibular origin (*n* = 124/309, 40.1%), which included 28 (9.1%) cases of Ménière’s disease, 68 (22.0%) cases of benign paroxysmal positional vertigo, 5 (1.6%) cases of vestibular neuronitis and 11 (3.6%) cases of other peripheral vertigo (S1 Table).

**Table 1:** Patient demographics.

Characteristic	Mean or percentage for the full analysis set
Age ± SD (mean), years	53.5 ± 15.1
Height ± SD (mean), cm	168.1 ± 9.4
Weight ± SD (mean), kg	74.5 ± 13.7
Sex (female:male), %	71.2:28.8
Race (White:Asian) %	99.7:0.3

SD, standard deviation

### Efficacy

#### Clinical response to betahistine treatment

The clinical response according to the change in SVVSLCRE level was rated as good, very good or excellent in 74.1% (226/305) of patients at end of treatment period (Table 2). The change in median SVVSLCRE level was found to be significant between baseline and Visit 2 (from Level IV to II; *p* < 0.001), and this reduction continued to the end of treatment (median of Level I) (S2 Table).

**Table 2:** Clinical response to betahistine treatment at the end of the treatment period.

Clinical response	Patients with clinical response (*N* = 305)^a^^,^^b^		
	*n*	%	95% CI
Excellent	43	14.1	10.4, 18.5
Very good	79	25.9	21.1, 31.2
Good	104	34.1	28.8, 39.7
Moderate	61	20.0	15.7, 24.9
No change	15	4.9	2.8, 8.0
Worsening	3	1.0	0.2, 2.8

^a^ Symptom severity was graded on the SVVSLCRE from Level I (0–2; absent vestibular vertigo) to Level V (8–10; very severe vestibular vertigo). Clinical response was graded based on the change from baseline to end of treatment in SVVSLCRE level and was described as worsening, no change, or on a 4-point scale from moderate (reduced by 1 level) to excellent (reduced by 4 levels);

^b^For 60-day treatment group.

CI, confidence interval; SVVSLCRE, Scale for Vestibular Vertigo Severity Level and Clinical Response Evaluation

#### Reductions in monthly vertigo attack frequency

The change in median monthly vertigo attack frequency (from 8.0 to 3.0 attacks per month) was significant between baseline and Visit 2 (*p* < 0.001), and this attack frequency continued to decrease to a median of 2.0 at end of treatment (Table 3). During the follow-up period, the median monthly vertigo attack frequency further decreased to 0.0 at both Months 1 and 2; this change from end of treatment was significant (*p* < 0.001). An exploratory sub-group analysis of patients grouped according to their different aetiologies showed that the reduction in monthly vertigo attack frequency during 2 months of follow-up was significant for a number of ICD-10 classes, including diseases of vestibular origin (*p* < 0.001), other headache syndromes (*p* = 0.017) and multiple sclerosis (*p* = 0.031 at 1-month follow-up) (S3 Table).

**Table 3:** Monthly vertigo attack frequency following treatment initiation.

Study visit	Monthly vertigo attack frequency	Change in monthly vertigo attack frequency			*N*^a^
	Median (Q1, Q3)	Change from Baseline, median (Q1, Q3)	Change from EOT, median (Q1, Q3)	*p* value	
Baseline	8 (4, 14)	**–**	**–**	**–**	305
Visit 2	3 (2, 5)	–4 (–8, –1)	**–**	< 0.001	304^b^
EOT	2 (0, 3)	–5 (–11, –2)	**–**	< 0.001	305
1-month FU	0 (0, 2)	**–**	–1 (–1, 0)	< 0.001	305
2-month FU	0 (0, 2)	**–**	–1 (–2, 0)	< 0.001	305

^a^ For 60-day treatment group;

^b^ *N* = 304 due to one patient having a combined Visit 2 and 3, which was only input as Visit 3

EOT, end of treatment; FU, follow-up; Q1, quartile 1; Q3, quartile 3

#### Physician and patient grading of overall clinical response

Physician grading of overall clinical response was assessed as either good or excellent in 75.7% (230/304) of patients at Visit 2, and this increased to 94.4% (288/305) of patients by end of treatment (Table 4). Patient grading of overall clinical response followed a similar trend; 76.3% (232/304) of patients assessed their overall clinical response as either good or excellent at Visit 2, and this increased to 95.4% (291/305) at end of treatment.

**Table 4:** Overall clinical response to betahistine treatment assessed by physicians and patients.

Overall clinical response	Assessed by physicians, *n* (%)		Assessed by patients, *n* (%)	
	Visit 2	End of treatment	Visit 2	End of treatment
Poor	3 (1.0)	2 (0.7)	3 (1.0)	4 (1.3)
Fair	71 (23.4)	15 (4.9)	69 (22.7)	10 (3.3)
Good	185 (60.9)	162 (53.1)	190 (62.5)	162 (53.1)
Excellent	45 (14.8)	126 (41.3)	42 (13.8)	129 (42.3)
**Total**^a^	**304**^b^	**305**	**304**^b^	**305**

^a^ For 60-day treatment group;

^b^ *N* = 304 due to one patient having a combined Visit 2 and 3, which was only inputted as Visit 3

#### Clinical improvement of vertigo-associated symptoms

Clinical improvement of specific vertigo-associated symptoms was considered either good or excellent by physicians or patients in up to 71.4% of patients at Visit 2, and in up to 90.5% of patients at end of treatment (Table 5; S4 Table). The highest proportion of good or excellent clinical improvement scores were observed for nausea (89.9–90.2%), vomiting (82.4–82.7%) and faintness (87.9–88.2%) at end of treatment.

**Table 5:** Improvement of vertigo-associated symptoms evaluated by physicians and patients.

Vertigo-associated symptom	Good or excellent clinical improvement, *n* (%)			
	Assessed by physician		Assessed by patient	
	Visit 2^a^	End of treatment^b^	Visit 2^a^	End of treatment^b^
Tinnitus	149 (49.0)	203 (66.6)	149 (49.0)	195 (63.9)
Hearing loss	130 (42.8)	177 (58.0)	127 (41.8)	176 (57.7)
Nausea	217 (71.4)	275 (90.2)	214 (70.4)	276 (90.5)
Vomiting	206 (67.8)	252 (82.6)	203 (66.8)	253 (83.0)
Faintness	214 (70.4)	269 (88.2)	215 (70.7)	270 (88.5)
Headache	191 (62.8)	240 (78.7)	185 (60.9)	238 (78.0)

^a^ *N* = 304 for the 60-day treatment group at Visit 2 (due to one patient having a combined Visit 2 and 3, which was only inputted as Visit 3);

^b^ *N* = 305 for the 60-day treatment group at end of treatment

#### Safety and tolerability

Only one AE was reported in this study, which was lack of drug efficacy leading to increased frequency of attacks of vertigo, nausea and heartburn. This led to the patient discontinuing the programme. No SAEs were reported in this study.

## Discussion

During this post-marketing observational study, betahistine (48 mg/day) was found to be effective for the treatment of vestibular vertigo. Betahistine treatment for up to 60 days was associated with significant reductions in vertigo severity (*p* < 0.001), as assessed by changes in SVVSLCRE level. Importantly, improvement continued beyond Day 30 of treatment, highlighting that clinicians may want to consider duration of betahistine treatment when trying to achieve maximal potential benefit.

Previous studies have demonstrated improvement in the severity of vertigo with betahistine treatment [9–13,15]. The current clinical evidence to support the effectiveness of betahistine should, however, be treated with caution. A 2016 Cochrane Review of randomised controlled trials of betahistine versus placebo found that although betahistine may have a positive effect on vertigo symptoms, there is significant variability between the results of the studies [16]. As a consequence, more rigorous methodology is required in future research.

Monthly vertigo attack frequency significantly decreased during the 2-month treatment period (*p* < 0.001) and continued to decrease during the 2-month follow-up, suggesting that a dose of betahistine 48 mg/day may have a lasting effect even after the cessation of treatment. Reductions in vertigo attack frequency were observed in previous studies that compared vertigo attack incidence between betahistine treatment and placebo [9–13] or baseline and 1–12 months of betahistine treatment [7,15]. In two of these studies, in which a dose of betahistine 48 mg/day was administered to most patients for 90 or 120 days, approximately 35% [12] or 50% [11] more patients, respectively, had reduced vertigo frequency compared with those on placebo. In studies where longer durations of betahistine treatment were assessed, at 24 weeks 91% more patients on treatment had no vertigo attacks compared with those on placebo [10], and at 3 months approximately 70% more patients than at baseline had no vertigo attacks [15]. These results, taken together with our observations, demonstrate that a daily dose of 48 mg/day may be effective for the reduction of vertigo attacks in the short and longer term.

Physician and patient grading of overall clinical response was good or excellent in most patients treated with betahistine in this study. Investigator and patient opinions of betahistine effectiveness have also been favourable in previous studies [9,10,12,13,17], supporting the use of betahistine as a good treatment option in daily clinical practice. Moreover, betahistine treatment was associated with good clinical improvement of vertigo-associated symptoms, with the largest improvements observed for nausea, vomiting and faintness. Previous comparable improvements in symptoms such as nausea and vomiting have been observed with betahistine treatment compared with placebo [9,13] or between baseline and up to 24 weeks of treatment [14,15]; thus, the results we observed reaffirm the effectiveness of betahistine for treatment of symptoms associated with vertigo.

Vertigo is a common condition that is associated with a considerable burden on health-related quality of life [2,4,5]. The positive patient and physician perceptions of betahistine treatment observed here suggest that betahistine may reduce symptoms that have a negative impact on patient wellbeing.

Benign paroxysmal positional vertigo is the most common form of peripheral vestibular vertigo [21,22], so as expected, this was the most frequent diagnosis according to ICD-10 sub-class of patients in VIRTUOSO (22.0%). The first choice of treatment for benign paroxysmal positional vertigo is currently canalith repositioning procedures, but these do not always successfully alleviate vertigo symptoms [21]. Betahistine was effective in improving vertigo symptoms in this study, which contained a considerable proportion of patients with benign paroxysmal positional vertigo. Other prospective evidence has demonstrated that treatment with betahistine and repositioning procedures resulted in greater improvement in vertigo symptoms compared with repositioning procedures alone [21,22]. Therefore, it would be interesting to conduct future studies comparing betahistine treatment to repositioning procedures, and assessing the efficacy of combining both treatments over a longer follow-up period.

No SAEs and only one AE were reported in 309 patients, confirming the well-established and favourable safety profile of betahistine administered both at 48 mg/day [7,10–12,15,17] and at lower doses [9,13,14].

The VIRTUOSO study does have some limitations, as with any observational study. For example, real-world evidence does not take into consideration potential sources of bias, missing data, the placebo effect and the natural fluctuations that occur in the different conditions. Future research, that is prospective and randomised in its design, is needed to provide evidence that the persistent beneficial effect of betahistine cannot be explained by natural fluctuations in symptoms alone. When reviewing the findings of the study, it is important to consider that vertigo is a highly subjective condition. The endpoints in the study were evaluated by a physician and/or the patient, and were subject to sources of bias (e.g. utilises the memory of the patients to recall severity of vertigo between visits). The study was only undertaken in two countries and, therefore, does not represent the global population; in addition, it may not provide a representative sample of patients from each country (e.g. enrolled patients were attending an outpatient clinic).

While acknowledging these limitations, the authors note that the outcomes of VIRTUOSO are comparable with those of other post-marketing studies assessing betahistine against placebo for vertigo treatment [9–13]. Furthermore, this study has provided real-world data suggesting that the effects of betahistine on vestibular compensation continue to be maintained for at least 2 months after treatment has ceased.

## Conclusion

This observational study found that treatment of vestibular vertigo with betahistine (dosed at 48 mg/day) appeared to be effective in reducing vertigo-associated symptoms in a routine outpatient clinical setting. The VIRTUOSO results showed that the effectiveness of betahistine treatment persisted for 2 months after cessation of treatment, which may suggest that betahistine may facilitate lasting vestibular compensation. Future controlled trials are required to confirm this observed compensatory effect. Betahistine was well tolerated when administered at 48 mg/day for 2 months, and should be considered as a good therapy option by physicians treating vertigo.

## Supporting information

S1 Table Patient diagnoses according to ICD-10 class. (DOC)

S2 Table SVVSLCRE level following treatment initiation. (DOC)

S3 Table Monthly vertigo attack frequency during follow-up period according to ICD-10 class. (DOC)

S4 Table Improvement of vertigo-associated symptoms evaluated by physicians and patients. (DOC)

S1 Fig Scale for Vestibular Vertigo Severity Level and Clinical Response Evaluation (SVVSLCRE). (PDF)

S2 Fig TREND statement checklist. (PDF)

S3 Fig Post-marketing observational program protocol (English). (PDF)

S4 Fig Post-marketing observational program protocol (Russian). (PDF)
