# Supplementary material for: Effectiveness of betahistine (48 mg/day) in patients with vestibular vertigo during routine practice: The VIRTUOSO study
Source: PLoS One. 2017 Mar 30;12(3):e0174114. doi: 10.1371/journal.pone.0174114 (PMC5373561; doi:10.1371/journal.pone.0174114)
Supplement: S1 Table — (DOC) [file pone.0174114.s001.doc]

S1 Table. Patient diagnoses according to ICD-10 class

| **ICD-10 class** | **Patients with diagnosis, *n* (%)** |
| --- | --- |
| G11.1 Early-onset cerebellar ataxia | 1 (0.3) |
| G11.2 Late-onset cerebellar ataxia | 1 (0.3) |
| G11.8 Other hereditary ataxias | 2 (0.6) |
| G24 Dystonia | 2 (0.6) |
| G35 Multiple sclerosis | 9 (2.9) |
| G37.0 Diffuse sclerosis | 1 (0.3) |
| G37.9 Demyelinating disease of central nervous system, unspecified | 1 (0.3) |
| G43 Migraine | 1 (0.3) |
| G43.1 Migraine with aura (classical migraine) | 5 (1.6) |
| G44.0 Cluster headache syndrome | 3 (1.0) |
| G44.1 Vascular headache, not elsewhere classified | 1 (0.3) |
| G44.2 Tension-type headache | 3 (1.0) |
| G44.3 Chronic post-traumatic headache | 7 (2.3) |
| G44.8 Other specified headache syndromes | 1 (0.3) |
| G45 Transient cerebral ischaemic attacks and related syndromes | 1 (0.3) |
| G45.0 Vertebro-basilar artery syndrome | 52 (16.8) |
| G45.8 Other transient cerebral ischaemic attacks and related syndromes | 1 (0.3) |
| G45.9 Transient cerebral ischaemic attack, unspecified | 1 (0.3) |
| G46 Vascular syndromes of brain in cerebrovascular diseases | 2 (0.6) |
| G46.0 Middle cerebral artery syndrome | 1 (0.3) |
| G46.8 Other vascular syndromes of brain in cerebrovascular diseases | 1 (0.3) |
| G47 Sleep disorders | 1 (0.3) |
| G47.0 Disorders of initiating and maintaining sleep (insomnias) | 1 (0.3) |
| G52.9 Cranial nerve disorder, unspecified | 3 (1.0) |
| G63.2 Diabetic polyneuropathy | 3 (1.0) |
| G70.0 Myasthenia gravis | 1 (0.3) |
| G90.9 Disorder of autonomic nervous system, unspecified | 4 (1.3) |
| G93.4 Encephalopathy, unspecified | 2 (0.6) |
| H80 Otosclerosis | 1 (0.3) |
| H80.1 Otosclerosis involving oval window, obliterative | 2 (0.6) |
| H80.2 Cochlear otosclerosis | 1 (0.3) |
| H80.9 Otosclerosis, unspecified | 1 (0.3) |
| H81.0 Ménière’s disease | 28 (9.1) |
| H81.1 Benign paroxysmal positional vertigo | 68 (22.0) |
| H81.2 Vestibular neuronitis | 5 (1.6) |
| H81.3 Other peripheral vertigo | 11 (3.6) |
| H81.4 Vertigo of central origin | 4 (1.3) |
| H81.8 Other disorders of vestibular function | 6 (1.9) |
| H81.9 Unspecified disorder of vestibular function | 2 (0.6) |
| H82 Vertiginous syndromes in diseases classified elsewhere | 1 (0.3) |
| H90.3 Sensorineural hearing loss, bilateral | 4 (1.3) |
| H90.4 Sensorineural hearing loss, unilateral with unrestricted hearing on the contralateral side | 2 (0.6) |
| H90.7 Mixed conductive and sensorineural hearing loss, unilateral with unrestricted hearing on the contralateral side | 1 (0.3) |
| H93.8 Disorder of ear, unspecified | 1 (0.3) |
| I65.0 Occlusion and stenosis of vertebral artery | 14 (4.5) |
| I65.1 Occlusion and stenosis of basilar artery | 1 (0.3) |
| I65.2 Occlusion and stenosis of carotid artery | 1 (0.3) |
| I65.3 Occlusion and stenosis of multiple and bilateral precerebral arteries | 1 (0.3) |
| I67.1 Cerebral aneurysm, nonruptured | 1 (0.3) |
| I67.2 Cerebral atherosclerosis | 6 (1.9) |
| I67.3 Progressive vascular leukoencephalopathy | 1 (0.3) |
| I67.4 Hypertensive encephalopathy | 11 (3.6) |
| I67.8 Other specified cerebrovascular diseases | 12 (3.9) |
| I67.9 Cerebrovascular disease, unspecified | 3 (1.0) |
| I69.3 Sequelae of cerebral infarction | 8 (2.6) |

ICD-10, International Classification of Diseases 10th Revision
