# Supplementary material for: Effectiveness of betahistine (48 mg/day) in patients with vestibular vertigo during routine practice: The VIRTUOSO study
Source: PLoS One. 2017 Mar 30;12(3):e0174114. doi: 10.1371/journal.pone.0174114 (PMC5373561; doi:10.1371/journal.pone.0174114)
Supplement: S2 Table — (DOC) [file pone.0174114.s002.doc]

S2 Table. SVVSLCRE level following treatment initiation

| **Study visit** | **SVVSLCRE level** | **Change in SVVSLCRE level from Baseline** | | ***N*^a^** |
| --- | --- | --- | --- | --- |
|  | **Median (Q1, Q3)** | **Median (Q1, Q3)** | ***p* value** |  |
| Baseline | 4 (3, 4) | **–** | **–** | 305 |
| Visit 2 | 2 (2, 3) | –1 (–2, –1) | < 0.001 | 304^b^ |
| End of treatment | 1 (1, 2) | –2 (–3, –1) | < 0.001 | 305 |

^a^ For 60-day treatment group;

^b^ *N* = 304 due to one patient having a combined Visit 2 and 3, which was only input as Visit 3
Q1, quartile 1; Q3, quartile 3; SVVSLCRE, Scale for Vestibular Vertigo Severity Level and Clinical Response Evaluation
