# Supplementary material for: Effectiveness of betahistine (48 mg/day) in patients with vestibular vertigo during routine practice: The VIRTUOSO study
Source: PLoS One. 2017 Mar 30;12(3):e0174114. doi: 10.1371/journal.pone.0174114 (PMC5373561; doi:10.1371/journal.pone.0174114)
Supplement: S3 Table — (DOC) [file pone.0174114.s003.doc]

S3 Table. Monthly vertigo attack frequency during follow-up period according to ICD-10 class

| **ICD-10 class** | **Monthly vertigo attack frequency, median (Q1, Q3)** | | **Change in monthly vertigo attack frequency from End of Treatment, median (Q1, Q3)** | | | |
| --- | --- | --- | --- | --- | --- | --- |
|  | **1-month FU** | **2-month FU** | **1-month FU** | ***p* value** | **2-month FU** | ***p* value** |
| G11 | 3.0 (3.0, 6.5) | 2.5 (2.0, 5.5) | –2.0 (–5.5, 0.0) | 0.500 | –3.5 (–6.5, –1.0) | 0.250 |
| G24 | 1.5 (0.0, 3.0) | 1.5 (0.0, 3.0) | 0.0 (–1.0, 1.0) | 1.000 | 0.0 (–1.0, 1.0) | 1.000 |
| G35 | 1.0 (0.0, 2.0) | 1.0 (0.0, 3.0) | –1.0 (–1.0, 0.0) | 0.031 | 0.0 (–1.0, 0.0) | 0.250 |
| G37 | 3.0 (2.0, 4.0) | 2.0 (2.0, 2.0) | –2.0 (–3.0, –1.0) | 0.500 | –3.0 (–3.0, –3.0) | 0.500 |
| G43 | 0.0 (0.0, 2.0) | 0.0 (0.0, 2.0) | 0.0 (0.0, 0.0) | 1.000 | 0.0 (–1.0, 0.0) | 0.500 |
| G44 | 0.0 (0.0, 3.0) | 0.0 (0.0, 2.0) | –1.0 (–2.0, 0.0) | 0.015 | –1.0 (–4.0, 0.0) | 0.017 |
| G45 | 0.0 (0.0, 1.0) | 0.0 (0.0, 1.0) | 0.0 (**–**1.0, 0.0) | 0.005 | 0.0 (**–**1.0, 0.0) | < 0.001 |
| G46 | 2.0 (1.0, 6.0) | 3.0 (0.5, 6.5) | 0.0 (0.0, 0.0) | **–** | –0.5 (**–**1.5, 1.5) | 1.000 |
| G47 | 2.0 (2.0, 2.0) | 2.0 (1.0, 3.0) | 0.0 (0.0, 0.0) | **–** | 0.0 (**–**1.0, 1.0) | 1.000 |
| G52 | 1.0 (0.0, 1.0) | 0.0 (0.0, 1.0) | –1.0 (**–**1.0, –1.0) | 0.250 | –1.0 (**–**2.0, –1.0) | 0.250 |
| G63 | 10.0 (0.0, 10.0) | 8.0 (0.0, 10.0) | 0.0 (**–**1.0, 0.0) | 1.000 | –1.0 (**–**2.0, 0.0) | 0.500 |
| G70 | 3.0 (3.0, 3.0) | 3.0 (3.0, 3.0) | –2.0 (–2.0, –2.0) | 1.000 | –2.0 (–2.0, –2.0) | 1.000 |
| G90 | 1.0 (0.5, 1.0) | 0.0 (0.0, 0.0) | –1.0 (–1.0, –1.0) | 0.125 | –2.0 (–2.0, –1.5) | 0.125 |
| G93 | 0.5 (0.0, 1.0) | 2.0 (1.0, 3.0) | –1.5 (–3.0, 0.0) | 1.000 | 0.0 (0.0, 0.0) | **–** |
| H80 | 0.0 (0.0, 0.0) | 0.0 (0.0, 0.0) | –1.0 (–1.0, 0.0) | 0.250 | –1.0 (–1.0, 0.0) | 0.250 |
| H81 | 0.0 (0.0, 1.0) | 0.0 (0.0, 1.0) | –1.0 (–2.0, 0.0) | < 0.001 | –1.0 (–2.0, 0.0) | < 0.001 |
| H82 | 1.0 (1.0, 1.0) | 0.0 (0.0, 0.0) | –2.0 (–2.0, –2.0) | 1.000 | –3.0 (–3.0, –3.0) | 1.000 |
| H90 | 0.0 (0.0, 0.0) | 0.0 (0.0, 0.0) | 0.0 (0.0, 0.0) | 1.000 | 0.0 (0.0, 0.0) | 1.000 |
| H93 | 3.0 (3.0, 3.0) | 3.0 (3.0, 3.0) | 1.0 (1.0, 1.0) | 1.000 | 1.0 (1.0, 1.0) | 1.000 |
| I65 | 1.0 (0.0, 4.0) | 1.0 (0.0, 4.0) | 0.0 (–1.0, 0.0) | 0.563 | 0.0 (–1.0, 0.0) | 0.844 |
| I67 | 1.0 (1.0, 3.0) | 2.0 (1.0, 2.0) | –1.0 (–2.0, 0.0) | 0.001 | –1.0 (–2.0, 1.0) | 0.058 |
| I69 | 2.0 (0.0, 4.5) | 1.0 (0.0, 4.0) | –1.0 (–2.0, 0.0) | 0.063 | –1.5 (–3.0, 0.0) | 0.063 |

FU, follow-up; ICD-10, International Classification of Diseases 10th Revision
