# Supplementary material for: Effectiveness of betahistine (48 mg/day) in patients with vestibular vertigo during routine practice: The VIRTUOSO study
Source: PLoS One. 2017 Mar 30;12(3):e0174114. doi: 10.1371/journal.pone.0174114 (PMC5373561; doi:10.1371/journal.pone.0174114)
Supplement: S4 Table — (DOC) [file pone.0174114.s004.doc]

S4 Table. Improvement of vertigo-associated symptoms evaluated by physicians and patients

| **Vertigo-associated symptom** | **Clinical improvement assessed by physician,^a^ *n* (%)** | | | | | | | | | |
| --- | --- | --- | --- | --- | --- | --- | --- | --- | --- | --- |
|  | **N/A** | | **Poor** | | **Fair** | | **Good** | | **Excellent** | |
|  | **V2** | **EOT** | **V2** | **EOT** | **V2** | **EOT** | **V2** | **EOT** | **V2** | **EOT** |
| Tinnitus | 79 (26.0) | 73 (23.9) | 13 (4.3) | 4 (1.3) | 63 (20.7) | 25 (8.2) | 108 (35.5) | 112 (36.7) | 41 (13.5) | 91  (29.8) |
| Hearing loss | 104 (34.2) | 89 (29.2) | 22 (7.2) | 17 (5.6) | 48 (15.8) | 22 (7.2) | 83 (27.3) | 80 (26.2) | 47 (15.5) | 97 (31.8) |
| Nausea | 10 (3.3) | 11 (3.6) | 3 (1.0) | 4 (1.3) | 74 (24.3) | 15 (4.9) | 157 (51.6) | 114 (37.4) | 60 (19.7) | 161 (52.8) |
| Vomiting | 53 (17.4) | 38 (12.5) | 5 (1.6) | 3 (1.0) | 40 (13.2) | 12 (3.9) | 106 (34.9) | 80 (26.2) | 100 (32.9) | 172 (56.4) |
| Faintness | 17 (5.6) | 11 (3.6) | 2 (0.7) | 5 (1.6) | 71 (23.4) | 20 (6.6) | 168 (55.3) | 119 (39.0) | 46 (15.1) | 150 (49.2) |
| Headache | 54 (17.8) | 45 (14.8) | 5 (1.6) | 6 (2.0) | 54 (17.8) | 14 (4.6) | 144 (47.4) | 106 (34.8) | 47 (15.5) | 134 (43.9) |
| **Vertigo-associated symptom** | **Clinical improvement assessed by patient,^a^ *n* (%)** | | | | | | | | | |
|  | **N/A** | | **Poor** | | **Fair** | | **Good** | | **Excellent** | |
|  | **V2** | **EOT** | **V2** | **EOT** | **V2** | **EOT** | **V2** | **EOT** | **V2** | **EOT** |
| Tinnitus | 76 (25.0) | 73 (23.9) | 14 (4.6) | 4 (1.3) | 65 (21.4) | 33 (10.8) | 107 (35.2) | 107 (35.1) | 42 (13.8) | 88 (28.9) |
| Hearing loss | 103 (33.9) | 89 (29.2) | 19 (6.3) | 15 (4.9) | 55 (18.1) | 25 (8.2) | 79 (26.0) | 74 (24.3) | 48 (15.8) | 102 (33.4) |
| Nausea | 11 (3.6) | 12 (3.9) | 2 (0.7) | 3 (1.0) | 77 (25.3) | 14 (4.6) | 152 (50.0) | 109 (35.7) | 62 (20.4) | 167 (54.8) |
| Vomiting | 51 (16.8) | 36 (11.8) | 5 (1.6) | 3 (1.0) | 45 (14.8) | 13 (4.3) | 108 (35.5) | 87 (28.5) | 95 (31.3) | 166 (54.4) |
| Faintness | 15 (4.9) | 9 (3.0) | 2 (0.7) | 5 (1.6) | 72 (23.7) | 21 (6.9) | 164 (53.9) | 129 (42.3) | 51 (16.8) | 141 (46.2) |
| Headache | 53 (17.4) | 42 (13.8) | 5 (1.6) | 5 (1.6) | 61 (20.1) | 20 (6.6) | 136 (44.7) | 105 (34.4) | 49 (16.1) | 133 (43.6) |

^a^ *N* = 304 for the 60-day treatment group at Visit 2 (due to one patient having a combined Visit 2 and 3, which was only inputted as Visit 3); *N* = 305 for the 60-day treatment group at EOT
EOT, end of treatment; N/A, not applicable; V2, Visit 2
