# Supplementary figures and images for: Effectiveness of betahistine (48 mg/day) in patients with vestibular vertigo during routine practice: The VIRTUOSO study
Source: PLoS One. 2017 Mar 30;12(3):e0174114. doi: 10.1371/journal.pone.0174114 (PMC5373561; doi:10.1371/journal.pone.0174114)

Scale for Vestibular Vertigo Severity Level and Clinical Response Evaluation (SVVSLCRE)

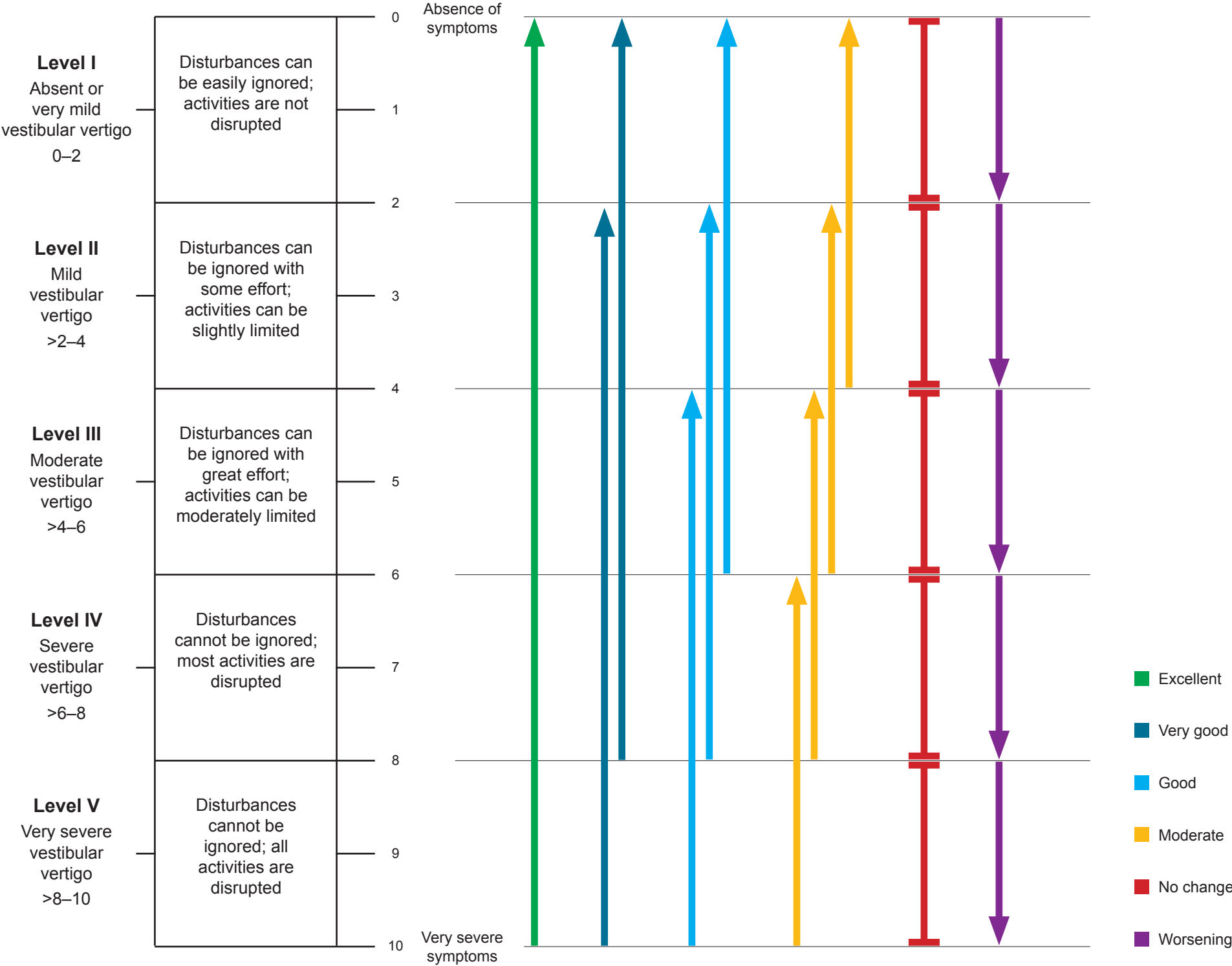

Supplement: S1 Fig — (PDF) [file pone.0174114.s005.pdf]
