# Supplementary material for: Effectiveness of betahistine (48 mg/day) in patients with vestibular vertigo during routine practice: The VIRTUOSO study
Source: PLoS One. 2017 Mar 30;12(3):e0174114. doi: 10.1371/journal.pone.0174114 (PMC5373561; doi:10.1371/journal.pone.0174114)
Supplement: S3 Fig — (PDF) [file pone.0174114.s007.PDF]

1.0 Title Page

Abbott Laboratories

Post-Marketing Observational Program Protocol (P13-972)

Amendment 1

Post-Marketing Observational Program of Betaserc® (Betahistine dihydrochloride) to Evaluate Effectiveness in Patients with Vestibular Vertigo in Routine Practice (VIRTUOSO)

Product Name: Betahistine dihydrochloride (Betaserc®)

Type of Study: Post-Marketing Observational Program

Date: December 10, 2012

Sponsor:

Abbott Laboratories
B. C. Metropolis
16A Building 1 Leningradskoe shosse,
Moscow, 125171 Russian Federation
Tel.: +7 495 258 4280
Fax: +7 495 258 4281

Abbott Ukraine
110 Zhylyanska str., 2nd floor
Kyiv, 01032 Ukraine
Tel.: +38 044 498 60 80
Fax: +38 044 498 60 81

1.1 List of Changes

The purpose of this amendment is to implement several format and administrative changes.

An itemized list of all changes made to the protocol under this amendment is included in Appendix.

2.0 Table of Contents

1.0 Title Page ... 1
1.1 List of Changes ... 2
2.0 Table of Contents ... 3
3.0 List of Abbreviations ... 5
4.0 Introduction ... 6
5.0 Rationale ... 8
6.0 Study Objectives ... 9
6.1 Primary Objective ... 9
6.2 Secondary Objectives ... 9
6.3 Primary Endpoint ... 9
6.4 Secondary Endpoints ... 11
7.0 Investigational Plan ... 14
7.1 Selection of Study Population ... 14
7.1.1 Inclusion Criteria ... 15
7.1.2 Exclusion Criteria ... 15
7.2 Number of Patients to be Enrolled ... 16
7.3 Investigator Selection Criteria ... 16
7.4 Program Duration ... 16
7.5 Program Conduct ... 17
7.5.1 Product Supply ... 21
7.5.2 Description of Activities ... 21
8.0 Adverse Events ... 23
8.1 Adverse Event Definition and Serious Adverse Event Categories ... 23
8.2 Severity ... 25
8.3 Relationship to Pharmaceutical Product ... 25
8.4 Serious Adverse Event Collection Period ... 25

8.5 Serious Adverse Event Reporting 26
8.6 Pregnancy Reporting 27
9.0 Ethics and Quality 27
10.0 Case Report Forms 28
11.0 Data Analysis Plans 29
12.0 Final Report and Publications 35
13.0 References 36
14.0 Appendix: Protocol Amendment 1: List of Changes 38

3.0 List of Abbreviations

AE	Adverse Event
eCRF	electronic Case Report Form
EDC	Electronic Data Capture
ENT	Ear, Nose and Throat
ICD-10	International Classification of Diseases 10th Revision
IEC	Independent Ethics Committee
MedDRA	Medical Dictionary for Regulatory Activities
SAE	Serious Adverse Event

4.0 Introduction

Vertigo is one of the most frequent symptoms in medical practice and may be revealed in more than 80 diseases. Vertigo can be found in approximately 5% of subjects within general practitioners' practice [1] and 10% within otolaryngologist patient population [2]. According to Yardley L. during an interview of 20 thousand men, aged between 18 up to 64 it was found that more than 20% of respondents suffered from vertigo within a month, 30% of them had vertigo within 5 years [3]. In 1000 respondents older 60 years, 30% of them reported that they have had vertigo have had vertigo lifelong [4]. Approximately every third patient who visit otolaryngologist suffer from different types of vertigo [5].

Vertigo, commonly referred as illusion of movement and spatial orientation, occurs due to imbalances of signals to the vestibular apparatus [6]. There are two types of vertigo: vestibular and non-vestibular vertigo. Vestibular vertigo can be caused by lesions of the central system (the vestibular nuclei of the brainstem, vestibular and vestibular communications centers of the brain) or peripheral parts (the vestibular nerve and labyrinth) of the vestibular system. Vestibular vertigo is a common problem affecting more than 5% of adults every year [6; 7]. Various factors like female sex, age, lower educational level, and other comorbid conditions, including tinnitus, depression, several cardiovascular diseases (hypertension dyslipidemia), other risk factors, are also observed to be associated with vestibular vertigo [21].

Vestibular vertigo may be classified by the level of affecting of vestibular analyzer. (central or peripheral part of vestibular system). Vestibular vertigo is connected with irritation of certain locations of vestibular analyzer and depending on the dysfunction location can be either peripheral or central [14]. If vertigo arises from the balance centers of the brain, it is usually milder, and has accompanying neurologic deficits, such as slurred speech, double vision or pathologic nystagmus [7; 11; 12, 13]. Brain pathology can cause a sensation of disequilibrium which is an off-balance sensation.

A number of conditions that involve the central nervous system may lead to vertigo including: migraine headaches, lateral medullary syndrome, multiple sclerosis [8; 9]. Horizontal optokinetic nystagmus is a symptom which can accompany vertigo.

Peripheral vestibular vertigo is characterized by episodic or persistent occurrence and objective feeling of swaying [10]. Meniere's disease is often responsible for episodes of vertigo that heighten in severity over a period of minutes, but last for several hours with a gradual improvement during another period of several hours. Vestibular neuronitis and episodes of labyrinthitis typically present with fairly abrupt onset vertigo (over a period of hours) with resolution of the acute phase over the next several days. Traumatic injuries or vascular infarction of the labyrinth cause a sudden onset of symptoms with a slow recovery from the acute phase over a period of days to weeks, often with residual effects over a period of several months [22].

Medical management of vestibular vertigo may range from home care therapy to other medical interventional options available based on the type of disease condition the patient is suffering from. Specific types of vestibular vertigo may require additional treatment and referral.

Betahistine

Betahistine was first registered in Canada in 1968 and, to date, is approved in >115 countries for Meniere's disease and the symptomatic treatment of vertigo. The increased amounts of histamine released from histaminergic nerve endings can stimulate H1 receptors, thus augmenting the direct agonistic effects of betahistine on these receptors. This would additionally explain the potent vasodilatory effects of betahistine in the inner ear, which are well documented [15].The reduction in the number of attacks is achieved by betahistine treatment via increase in blood flow to the inner ear. This reduces the increased endolymphatic pressure in the inner ear by achieving a balance between production and re-absorption of endolymph [16]. In addition, betahistine strongly demonstrates facilitation of vestibular compensation through various animal models by a clear action on the histaminergic system; wherein it increases the histamine turnover and

release by blocking pre-synaptic histamine H3 auto receptors (both in vestibular and tuberomammillary nuclei) [15].

In clinical practice in Russia and Ukraine, betahistine dihydrochloride is administered at a dosage of 24-48 mg per day orally either as monotherapy or as adjunctive therapy when current anti-vertigo therapy is not sufficient or not tolerated.

5.0 Rationale

The purposes of this international post-marketing observational program is to investigate effectiveness of betahistine dihydrochloride (Betaserc®) tablets and assess the course of vestibular vertigo in post-treatment period in population of Russia and Ukraine outpatients suffering from vestibular vertigo in pragmatic clinical settings. The special goal of this study is to evaluate the use of Betahistine dihydrochloride in the maximal recommended daily dose of 48 mg in accordance with the locally approved label.

As it is well known that 2 – 3 months betahistine dihydrochloride therapy may accelerate and facilitate vestibular compensation in patients with vestibular dysfunction [23, 24] this study is also intended to assess the course of vestibular vertigo after Betaserc® discontinuation in descriptive manner. Two month length of follow-up period was selected because it is considered as an adequate time-point of symptom back for the majority of underlying neurological and otolaryngological conditions (unpublished data from Russian and Ukrainian academic neurologists and otorhinolaryngologists communications).

Exploratory analyses of results from both participating countries may be expected to provide insights about the subjective circumstances of vestibular vertigo patients in a wider than usual range of gender, underlying ICD-10 diagnosis, national and cultural situations.

6.0 Study Objectives

6.1 Primary Objective

1. To assess the effectiveness of betahistine dihydrochloride tablets 48mg/day therapy in routine outpatient clinical settings in population of subjects with vestibular vertigo.

6.2 Secondary Objectives

1. To assess the overall clinical response and clinical response as improvement of vertigo associated symptoms over treatment period.
2. To assess the course of vestibular vertigo over the 2-month post-treatment follow-up period.
3. To describe underlying diagnosis according to ICD-10 that account for vestibular vertigo.

6.3 Primary Endpoint

1. Number and proportion of patients with clinical response at the end of observational treatment period that is determined based on the Scale for Vestibular Vertigo Severity Level and Clinical Response Evaluation (SVVSLCRE) (Figure 1 and Table 1) in the total program population.

The patients will evaluate vestibular vertigo severity at baseline and end of observational treatment period based on the SVVSLCRE.

Figure 1. Scale for Vestibular Vertigo Severity Level and Clinical Response Evaluation (SVVSLCRE).

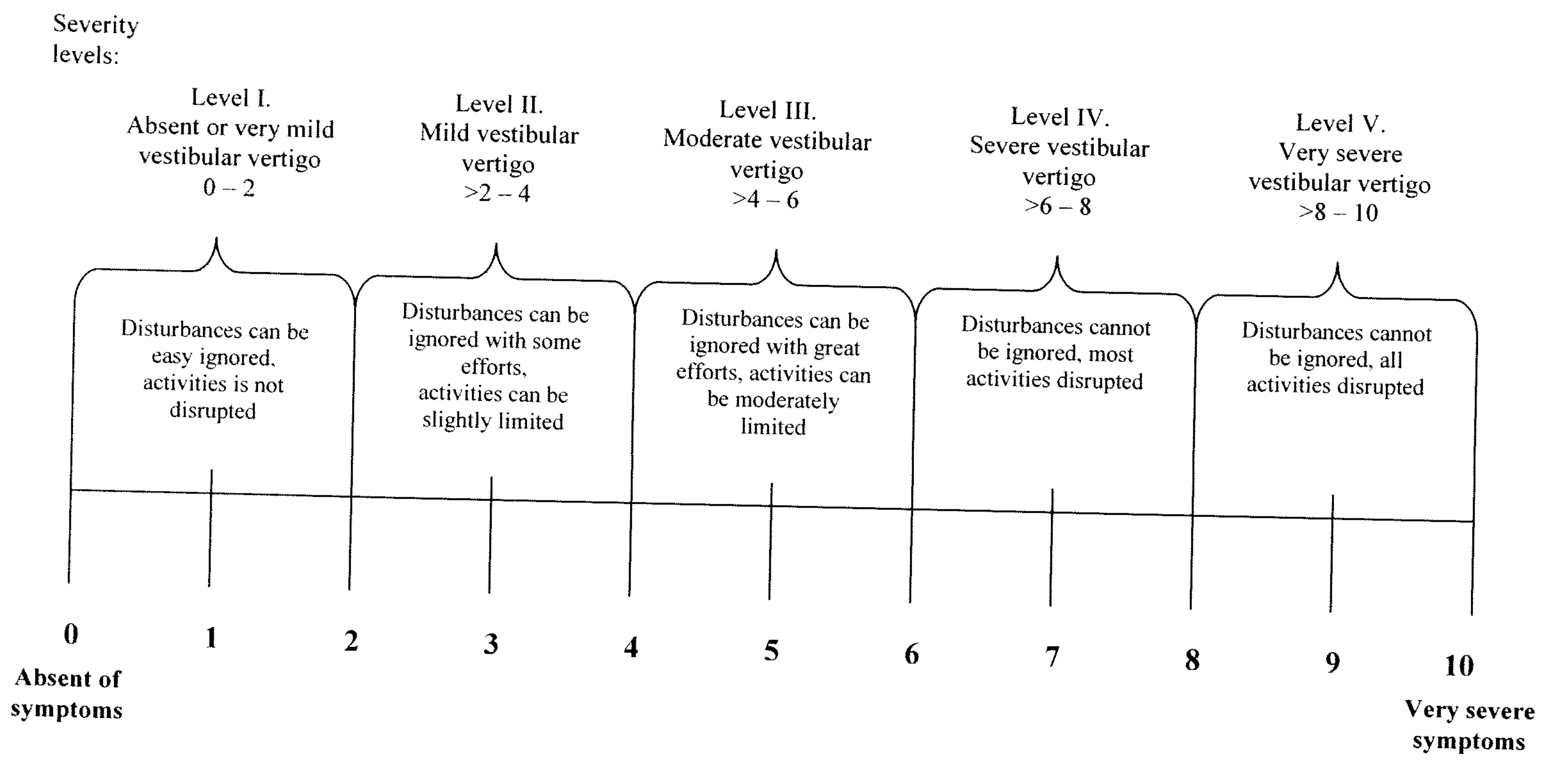

Table 1. Clinical responses define as a following:

Change of patient's clinical conditions of vestibular vertigo	*Shift direction based on the SVVSLCRE from Baseline to end of observational treatment period*
Worsening	All other shifts
No change	V to V; IV to IV; III to III; I to I
Moderate	V to IV; IV to III; III to II; II to I
Good	V to III; IV to II; III to I
Very good	V to II; IV to I
Excellent	V to I

6.4 Secondary Endpoints

1. Number and proportion of patients in each category of SVVSLCRE at baseline and end of observational treatment period in patients who completed betahistine treatment over a period less or equal 30 days and in patients who completed betahistine treatment over a period more than 30 days but less or equal 60 days.

2. Change of the patient's clinical conditions of vestibular vertigo assessed with SVVSLCRE from baseline to the end of observational treatment period in patients who completed betahistine treatment over a period less or equal 30 days and in patients who completed betahistine treatment over a period more than 30 days but less or equal 60 days.

3. Change of the patient's clinical conditions of vestibular vertigo assessed with SVVSLCRE from baseline to Visit 2 in patients who completed betahistine treatment over a period more than 30 days but less or equal 60 days.

4. Change of vestibular vertigo attacks frequency from baseline to the end of observational treatment period in patients who completed betahistine treatment over a period less or equal 30 days and in patients who completed betahistine treatment over a period more than 30 days but less or equal 60 days.

5. Change of vestibular vertigo attacks frequency from baseline to Visit 2 in patients who completed betahistine treatment over a period more than 30 days but less or equal 60 days.

6. Overall clinical response assessed by physician will be determined based on a four-point-scale, where 4 = excellent, 3 = good, 2 = fair, and 1 = poor. Proportion of patients in each category at the end of observational treatment period will be determined in patients who completed betahistine treatment over a period less or equal 30 days and in patients who completed betahistine treatment over a period more than 30 days but less or equal 60 days. Proportion of patients in each category at Visit 2 will be determined in patients who completed betahistine treatment over a period more than 30 days but less or equal 60 days.

7. Overall clinical response assessed by patient will be determined based on a four-point-scale, where 4 = excellent, 3 = good, 2 = fair, and 1 = poor. Proportion of patients in each category at the end of observational treatment period will be determined in patients who completed betahistine treatment over a period less or equal 30 days and in patients who completed betahistine treatment over a period more than 30 days but less or equal 60 days. Proportion of patients in each category at Visit 2 will be determined in patients who completed betahistine treatment over a period more than 30 days but less or equal 60 days.

8. Clinical response as improvement of vertigo associated symptoms (tinnitus, hearing loss, nausea, vomiting, faintness and headache) and evaluated by physician will be determined based on a four-point-scale, where 4 = excellent, 3 = good, 2 = fair, and 1 = poor. Proportion of patients in each category at the end of observational treatment period will be determined in patients who completed betahistine treatment over a period less or equal 30 days and in patients who completed betahistine treatment over a period more

than 30 days but less or equal 60 days. Proportion of patients in each category at Visit 2 will be determined in patients who completed betahistine treatment over a period more than 30 days but less or equal 60 days.

9. Clinical response as improvement of vertigo associated symptoms (tinnitus, hearing loss, nausea, vomiting, faintness and headache) and evaluated by patient will be determined based on a four-point-scale, where 4 = excellent, 3 = good, 2 = fair, and 1 = poor. Proportion of patients in each category at the end of observational treatment period will be determined in patients who completed betahistine treatment over a period less or equal 30 days and in patients who completed betahistine treatment over a period more than 30 days but less or equal 60 days. Proportion of patients in each category at Visit 2 will be determined in patients who completed betahistine treatment over a period more than 30 days but less or equal 60 days.

10. Change of vestibular vertigo attacks frequency from the end of observational treatment period to the end of 1st month of follow-up period in patients who completed betahistine treatment over a period less or equal 30 days and in patients who completed betahistine treatment over a period more than 30 days but less or equal 60 days.

11. Change of vestibular vertigo attacks frequency from the end of observational treatment period to the end of 2nd month of follow-up period (end of follow-up period) in patients who completed betahistine treatment over a period less or equal 30 days and in patients who completed betahistine treatment over a period more than 30 days but less or equal 60 days.

12. Specific underling ICD-10 diagnosis account for Vestibular Vertigo. Proportion of patients in each category at Visit 1 will be determined in total study population.

7.0 Investigational Plan

The program is designed as a prospective, multicentre, observational, non-interventional, non-randomized, non-controlled, single arm, Post-Marketing program.

7.1 Selection of Study Population

This is a prospective, multinational, non-comparative, Post-Marketing observational program in patients whom betahistine dihydrochloride (Betaserc®) tablets were prescribed in the usual manner at the maximal recommended daily dose of 48 mg in accordance with the locally approved label (prescribing information/summary of product characteristics). Over a program period physician is free to adjust Betaserc® dose according to country approved label.

Adult patients who can be treated with betahistine dihydrochloride as per the locally approved label (vestibular vertigo) will be enrolled in the study. This program does not include patients who required betahistine therapy, according to physician's decision, more than 2 consecutive months.

The dosage 48 mg will be under investigation and can be divided over the day as following:

8 and 16 mg tablets

8 mg tablets	16 mg tablets
2 tablets<br>3 times/day	1 tablet<br>3 times/day

24 mg tablets
The dosage for adults is 48 mg divided over the day.

24 mg tablets
1 tablet<br>2 times/day

The assignment of the patient to betahistine dihydrochloride is not decided in advance by this protocol but falls within current practice. The prescription of betahistine

dihydrochloride (Betaserc®) is clearly separated from the decision to include the patient in this program. No additional procedures (other than standard of care) shall be applied to the patients.

The patients must provide written consent to the Investigator to use and/or disclose personal and/or health data before entry into this program.

7.1.1 Inclusion Criteria

1. Male or female with age ≥ 18 years.

2. Patients with vestibular vertigo of known or unknown origin, and for whom the physician has decided to prescribe 48 mg of betahistine dihydrochloride (Betaserc®) in accordance with locally approved label.

3. Patients who are willing and able to provide authorization to the investigator to use and/or disclose personal and/or health data.

4. Patients who started betahistine dihydrochloride (Betaserc®) therapy not more than 5 days priory to sign Patient Authorization (Consent) for Use/Disclosure of Data.

7.1.2 Exclusion Criteria

1. Patients with any condition which, in the opinion of the Investigator, makes the patient unsuitable for inclusion based on clinical judgment.

2. Labeled contraindications of betahistine dihydrochloride (Betaserc®) treatment.

3. Patients with middle or inner ear infection.

4. Patient with psychiatric disorders, significant neurological disorder or spinal cord damage.

5. Patients receiving any other agents for peripheral vestibular vertigo such as diuretics, transtympanic gentamycin, cinnarizine, competitive antagonist of histamine, blocking H1-histamine receptors.

6. Patients undergoing ear surgery for vestibular disorders.

7. Female patients who are pregnant, planning to become pregnant or are breast-feeding.

8. Inability to comply with the protocol requirements.

9. Participation in any other clinical trial within last 30 days.

10. Patients who had previous betahistine therapy within 4 weeks of start of current betahistine dihydrochloride (Betaserc®) treatment course.

7.2 Number of Patients to be Enrolled

A total of 310 patients will be enrolled in this program from approximately 10-35 centers in Russia and Ukraine.

7.3 Investigator Selection Criteria

Criteria that will be used to select investigators to participate in the program:

1) Specialized in otorhinolaryngologists (ENT specialists) or neurologists

2) Ability to appropriately conduct the observational program

3) Availability of the outpatient population with vestibular vertigo

7.4 Program Duration

Each patient will be observed approximately 2 months within the observational treatment period and approximately 2 months within follow-up period for the evaluation during the course of vestibular vertigo after Betaserc® treatment discontinuation. Based on underling ICD-10 diagnosis and the clinical judgment of the physician, duration of treatment period

can be less than 2 months or can continue after termination of program's observational treatment period. However, only patients who finish betahistine dihydrochloride (Betaserc®) within 1 - 2 months of therapeutic period will be observed for the assessment of the course of vestibular vertigo within 2 months of follow-up period.

7.5 Program Conduct

The patient visits will be scheduled based on routine practice of the physician. 60 days of observational treatment period has been chosen as a length of time sufficient to generate useful effectiveness data. Follow-up visits for this program has been recommended keeping in mind the treating physician's usual pattern of follow-up consultations.

To optimize data collection, three visits are indicated within approximately 2 months of observational treatment period and two visits within approximately 2 months of follow-up period:

I. Observational treatment period

Visit 1: Baseline/Start of therapy

Visit 2: up to 30th day from the first dose of Betaserc®

Visit 3: up to 60th day from the first dose of Betaserc®, End of observation treatment period / Early Termination

II. Evaluation of the course of vestibular vertigo in post-treatment period, follow-up period (only for patients who finished betahistine dihydrochloride (Betaserc®) 1 - 2 months treatment period)

Visit 4: 30th day from the last dose of Betaserc®

Visit 5: 60th day from the last dose of Betaserc®, End of follow-up period

In the post-treatment period, the site personnel will contact the patients personally or by phone on 30 days after the last dose of Betaserc® to assess any SAE that may have started after the end of therapeutic observational period and also to follow-up on any SAE that were continuing from the time of last contact with patient.

Figure 2 below presents a schematic of the program plan and Table 2 below summarizes the various activities to be conducted at each of these visits.

Figure 2: Overall Investigational Plan of Study

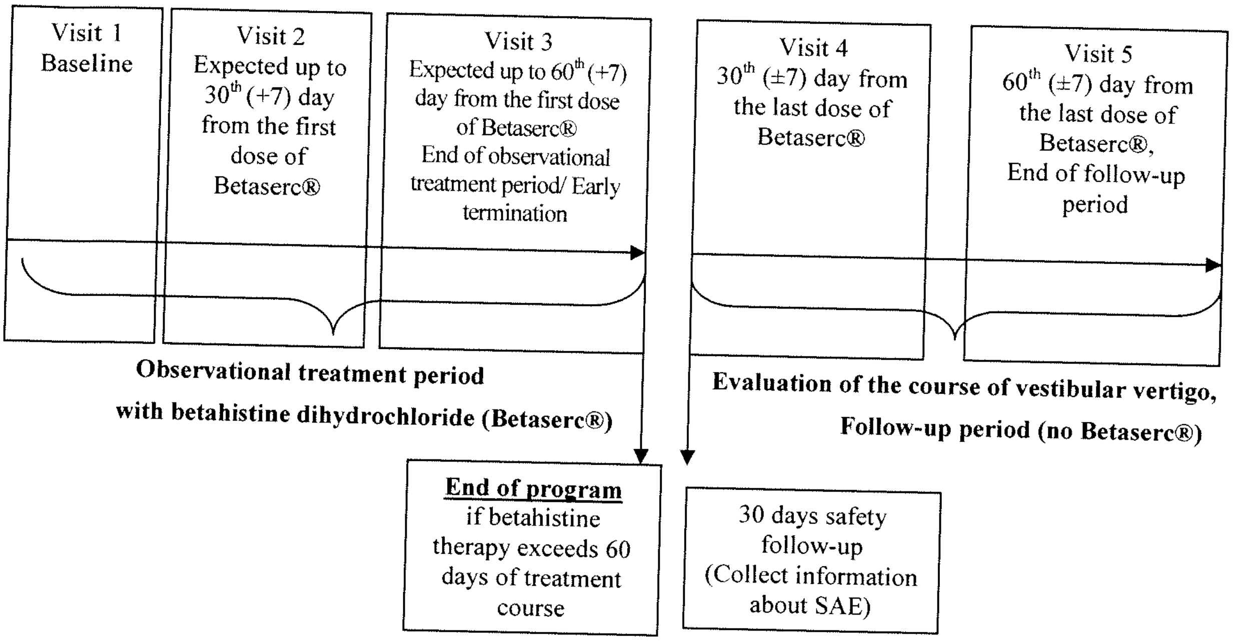

Table 2 Program Activities

Weeks of Treatment		Observational treatment period[1]		Evaluation of the course of vestibular vertigo in follow-up period	
	Visit 1	Visit 2	Visit 3	Visit 4	Visit 5
		Expected up to 30th day from the first dose of Betaserc®[2]	Expected up to 60th day from the first dose of Betaserc®[2]	30th (±7) day from the last dose of Betaserc®	60th (±7) day from the last dose of Betaserc®
	Screening/ Baseline		End of observational treatment period[3,4]/Early termination		End of follow-up period
Patient Authorization (Consent) for Use/Disclosure of Data	X				
Inclusion / exclusion criteria	X				
Register date of start of betahistine dihydrochloride (Betaserc®) treatment	X				
Demographics[5]	X				
Medical history	X				
Concomitant medication	X	X	X	X	X
Physical Exam (including vitals[6])	X				
Specific underlying ICD-10 diagnosis account for Vestibular Vertigo	X				
Number of monthly vertigo attacks	X	X	X	X	X
Scale for Vestibular Vertigo Severity Level and Clinical Response Evaluation[7]	X	X	X		
Overall clinical response and clinical response as improvement of vestibular vertigo associated symptoms based on four-point scales[8]		X	X		
SAE monitoring[9]	X	X	X	X	

1 This program does not include patients who required betahistine therapy, according to physician's decision, more than 60 days.

2 Actual date of visit may be up to 7 days from the last betahistine dose, otherwise proceed to Visit 4.

[3] This coincides with Visit 2 in case patient had completed betahistine treatment period lasting 30 days or less. In case physician decides to continue betahistine therapy beyond 30 days, Visit 3 will be a separate visit from Visit 2.

[4] For patients whom physician decides to continue betahistine therapy beyond 60 days treatment course, no follow up will be done and end of observational treatment period will denote end of program.

[5] Includes sex, age, race (white, black, asian, other), weight and height

[6] Heart rate, blood pressure (systolic and diastolic)

[7] for more detailed information about Scale for Vestibular Vertigo Severity Level and Clinical Response Evaluation please see section 6.3, Figure 1 and Table 1.

[8] Four-point-scale, where 4 = excellent, 3 = good, 2 = fair, and 1 = poor.

[9] 30 days after end of program treatment or early termination, a telephone call will be made to enquire about development of any SAE. If there is a positive feedback, relevant medical history and adverse event details will be collected.

7.5.1 Product Supply

As this is a Post-Marketing observational program, the Sponsor will not be involved in the product supply since the Betaserc® is being used according to the approved marketing authorization in the respective countries and is to be prescribed by the physician under usual and customary practice of physician prescription.

7.5.2 Description of Activities

Visit 1, Baseline / Start of therapy:

Following activities will be performed:

- Sign and Date of Patient Authorization (Consent) for Use/Disclosure of Data.
- Check inclusion/exclusion criteria
- Collect demographic data
- Register date of start of betahistine dihydrochloride (Betaserc®) treatment (can differ from enrollment date, please see section 7.1.1)
- Register concomitant medication

- Collect medical history
- Allocation of patient identification number
- Performed routine physical examination including vitals (heart rate, systolic and diastolic blood pressure)
- Collect specific underling ICD-10 diagnosis account for Vestibular Vertigo
- Register of the number of vertigo attacks for the previous month
- Evaluate patient's clinical conditions based on SVVSLCRE
- SAE monitoring

Visit 2, up to 30th day from the first dose of Betaserc®:

The following procedures will be performed:

- Register concomitant medication
- Evaluate patient's clinical conditions based on SVVSLCRE
- Register of the number of vertigo attacks for the previous month
- Assess overall clinical response and clinical response as improvement of vestibular vertigo associated symptoms based on four-point scale
- SAE monitoring

Visit 3, up to 60th day from the first dose of Betaserc®, End of Observational Treatment Period / Early Termination:

The following procedures will be performed:

- Register concomitant medication
- Evaluate patient's clinical conditions based on SVVSLCRE
- Register of the number of vertigo attacks for the previous month

- Assess overall clinical response and clinical response as improvement of vestibular vertigo associated symptoms based on four-point scale
- SAE monitoring

Visit 4, 30th (±7) day from the last dose of Betaserc®:

- Register concomitant medication
- Register of the number of vertigo attacks for the previous month
- SAE monitoring

Visit 5, 60th (±7) day from the last dose of Betaserc®:

- Register concomitant medication
- Register of the number of vertigo attacks for the previous month

8.0 Adverse Events

8.1 Adverse Event Definition and Serious Adverse Event Categories

An adverse event (AE) is defined as any untoward medical occurrence in a patient, which does not necessarily have a causal relationship with their treatment. An adverse event can therefore be any unfavorable and unintended sign (including an abnormal laboratory finding), symptom, or disease temporally associated with the use of a medicinal product, whether or not the event is considered causally related to the use of the product.

Such an event can result from use of the drug as stipulated in the labeling, as well as from accidental or intentional overdose, drug abuse, or drug withdrawal. Any worsening of a pre-existing condition or illness is considered an adverse event.

If an adverse event meets any of the following criteria, it is considered a **serious adverse event (SAE)**:

**Death of Subject:**	An event that results in the death of a subject.
**Life-Threatening:**	An event that, in the opinion of the investigator, would have resulted in immediate fatality if medical intervention had not been taken. This does not include an event that would have been fatal if it had occurred in a more severe form.
**Hospitalization:**	An event that results in an admission to the hospital for any length of time. This does not include an emergency room visit or admission to an outpatient facility.
**Prolongation of Hospitalization:**	An event that occurs while the study subject is hospitalized and prolongs the subject's hospital stay.
**Congenital Anomaly:**	An anomaly detected at or after birth, or any anomaly that results in fetal loss.
**Persistent or Significant Disability/Incapacity:**	An event that results in a condition that substantially interferes with the activities of daily living of a study subject. Disability is not intended to include experiences of relatively minor medical significance such as headache, nausea, vomiting, diarrhea, influenza, and accidental trauma (e.g., sprained ankle).
**Important Medical Event Requiring Medical or Surgical Intervention to Prevent Serious Outcome:**	An important medical event that may not be immediately life-threatening or result in death or hospitalization, but based on medical judgment may jeopardize the subject and may require medical or surgical intervention to prevent any of the outcomes listed above (i.e., death of subject, life threatening, hospitalization, prolongation of hospitalization, congenital anomaly, or persistent or significant disability/incapacity). Additionally, any elective or spontaneous abortion or stillbirth is considered an important medical event. Examples of such events include allergic bronchospasm requiring intensive treatment in an emergency room or at home, blood dyscrasias or convulsions that do not result in inpatient hospitalization, or the development of drug dependency or drug abuse.

8.2 Severity

The physician will use the following definitions to rate the severity for any adverse event being collected as an endpoint/data point in the study and for all serious adverse events.

Mild: The adverse event is transient and easily tolerated by the subject.

Moderate: The adverse event causes the subject discomfort and interrupts the subject's usual activities.

Severe: The adverse event causes considerable interference with the subject's usual activities and may be incapacitating or life threatening.

8.3 Relationship to Pharmaceutical Product

The investigator will use the following definitions to assess the relationship of the adverse event to the use of study drug:

Reasonable Possibility An adverse event where there is evidence to suggest a causal relationship between the study drug and the adverse event.

No Reasonable Possibility An adverse event where there is no evidence to suggest a causal relationship between the study drug and the adverse event.

If an investigator's opinion of no reasonable possibility of being related to study drug is given, an alternate etiology must be provided by the investigator for the adverse event.

8.4 Serious Adverse Event Collection Period

Serious adverse events will be reported to Abbott from the time the physician obtains the patient's authorization to use and disclose information (or the patient's informed consent) until 30 days following the intake of the last dose of physician-prescribed treatment.

8.5 Serious Adverse Event Reporting

In the event of a serious adverse event, the physician will:

- For events from patients using and Abbott product - notify the Abbott contact person identified below within 24 hours of the physician becoming aware of the event.

Contact information of Abbott representatives:

Russia:

Affiliate Safety Representative:

Alexey Ryakhin, MD
Pharmacovigilance Manager, EPD

Abbott Laboratories
B. C. Metropolis
16A Building 1 Leningradskoe shosse,
Moscow, 125171 Russian Federation
Tel: +7 495 258 4280
Mob: +7 926 101 49 64
Fax: +7 495 258 4281
aleksey.riahin@abbott.com

Affiliate Safety Representative (back-up):

Anton Pisarikhin
Pharmacovigilance specialist, EPD

Abbott Laboratories
B. C. Metropolis
16A Building 1 Leningradskoe shosse,
Moscow, 125171 Russian Federation
Tel: +7 495 258 4280
Mob: +79261979966
Fax: +7 495 258 4281
anton.pisarihin@abbott.com

Ukraine:

Affiliate Safety Representative

Mr. Oleksandr Molodetskyi
Affiliate Safety Representative

Abbott Ukraine,
110 Zhylyanska Str., 2nd floor
Kyiv, 01032 Ukraine

Tel: +380 44 498-60-80
Fax: +380 44 498-60-84

Affiliate Safety Representative (back-up)

Mrs. Anna Ivanova
Affiliate Safety Representative

Abbott Ukraine,
110 Zhylyanska Str., 2nd floor
Kyiv, 01032 Ukraine

Tel: +380 44 498-60-80
Fax: +380 44 498-60-84

8.6 Pregnancy Reporting

Information regarding a pregnancy occurrence in a study subject and the outcome of the pregnancy will be collected.

Pregnancy in a study subject is not considered an adverse event. However, any complication of pregnancy, (such as elective or spontaneous abortion, stillbirth, congenital anomaly etc.) is considered a serious adverse event and must be reported to Abbott within 24 hours of the site becoming aware of the event.

In the event of a pregnancy occurrence in the patient, the physician will notify the Abbott contact person identified in Section 8.5 within 24 hours of the physician becoming aware of the pregnancy.

9.0 Ethics and Quality

Patient's written authorization to use and/or disclose personal and/or health data must be obtained prior to enrolling each patient in the program and patients not willing to provide such written authorization will not be included in the program. However, all reasonable efforts will be made in order to avoid subject identifying information (such as name, address, etc).

Russia: the protocol and relevant program documents will be submitted for review and approval of Regulatory Authorities and Central and/or local (if applicable) Independent Ethics Committee(s) (IEC) and patient enrollment will start only after obtaining written approval from the IEC.

Ukraine: the protocol and relevant study documents will be submitted for review and approval to State Expert Center of Ministry of Health of Ukraine by Abbott. It is the responsibility of each investigator to obtain study approval (if applicable) of Local IEC before initiating the study, if applicable.

All data will be captured and handled in such a way so as to not reveal identity of individual patients and hence patient confidentiality will be maintained at all times.

The physician/investigator will be responsible for ensuring that a quality control and quality assurance system is in place to ensure that the program is conducted and data generated, documented, and reported in compliance with the protocol, accepted standards of Good Clinical Practice, and any applicable local laws and regulations.

The Abbott representative can monitor the case report forms after completion in EDC. Any discrepancies will be communicated to site for clarification. Any necessary corrections will be made to the database per applicable EDC procedures.

10.0 Case Report Forms

For this program electronic Case report forms (eCRF) will be used. eCRF will be completed for each subject enrolled in this program. These forms will be used to transmit and collect the program data for Abbott (or a CRO for data management) and regulatory authorities, as applicable. Electronic CRFs will be set by Abbott designee as web based site. All eCRFs must be legible and data must be entered by trained and authorized person only. eCRF training will be performed for site staff during site initiation. eCRF tracks all changes to the entered data automatically. Data discrepancies detected by eCRF will be addressed to investigator. All information entered in the eCRFs must also be reflected in the subject source documents.

The investigator will store subject data in his/her source documents in accordance to his/her praxis routine. These subject files will serve as source data for the program. The principal investigator will review the eCRFs for completeness and accuracy and sign each subject's set of eCRFs by electronic signature where indicated. The eCRFs will be reviewed periodically for completeness, legibility and acceptability by Abbott personnel (or their representatives).

11.0 Data Analysis Plans

General Principles

The principal features of the statistical analysis of the data are described in this section. Since this is an open-label, non-randomized trial, the analyses will primarily involve the generation of descriptive summary statistics. Quantitative variables will be summarized with number of patients, mean, standard deviation, median, 1st quartile, 3rd quartile, minimum and maximum values. Qualitative variables will be summarized with the number and proportion of patients in each category. In addition, two-sided 95% confidence intervals will be generated for parameter estimates. Graphs may be generated for visual interpretation of the results, as appropriate.

Data Analysis Set

All Patients Set will include all patients who signed patient authorization form to participate in the study. This population will be used for presenting disposition of subjects.

Full Analysis Set will include those patients who attended Visit 1 and who were administered at least one dose of betahistine dihydrochloride (Betaserc®), as assessed in the CRF. This population will be used for the reporting of effectiveness results.

Summary of Demographic and Baseline Characteristics

Demographic and baseline characteristics will be summarized with descriptive statistics described above and presented for total study population as well as for each involved country – Russia and Ukraine.

- Age will be summarised with number of patients, mean, standard deviation, median, 1st quartile, 3rd quartile, minimum and maximum values. Proportion of patient with age greater or less and equal to median will be presented.

- Sex and race will be summarized with number and proportion of patients in each category.
- Weight and height will be used for calculation of body mass index. Body mass index will be summarised with number of patients, mean, standard deviation, median, 1st quartile, 3rd quartile, minimum and maximum values.
- Findings of concomitant medication will be summarized with number and proportion of patients in each category.
- Findings of medical history will be summarized with number and proportion of patients in each category.
- Findings of physical examination will be summarized with number and proportion of patients in each category. Heart rate, blood pressure (systolic and diastolic), will be summarised with number of patients, mean, standard deviation, median, 1st quartile, 3rd quartile, minimum and maximum values. Proportion of patients with potentially clinically significant vital signs findings will be summarized with number and proportion of patients in each category.

Criteria for identifying potentially clinically significant vital signs findings presented below:

Criteria for Identifying Potentially Clinically Significant Vital Signs Findings

Variable (units)		Range	
Blood Pressure	Systolic Blood Pressure (mmHg)	80	160
	Diastolic Blood Pressure (mmHg)	50	100
Heart rate (bpm)		40	150

- Specific underling ICD-10 diagnosis account for Vestibular Vertigo will be summarized with number and proportion of patients in each category.

Analysis of Primary Endpoint

1. Number and proportion of patients with clinical response at the end of observational treatment period that is determined based on the SVVSLCRE. The normal approximation two-sided 95% confidence interval for binominal proportion will be generated.

Secondary Endpoints

Analysis of secondary endpoints will be done only for supporting evidence without any intention for a claim of treatment benefit for the secondary endpoints. Therefore, p-values and confidence intervals for secondary endpoints are purely for descriptive purposes and not for drawing inferences.

1. Number and proportion of patients in each category of SVVSLCRE at baseline and the end of observational treatment period will be presented for patients who completed betahistine treatment over a period less or equal 30 days and for patients who completed betahistine treatment over a period more than 30 days but less or equal 60 days.

2. Change of the patient's clinical conditions of vestibular vertigo assessed with SVVSLCRE from baseline to the end of observational treatment period will be presented with median, 1st quartile and 3rd quartile. Comparison between baseline and the end of observational treatment period will be made using Wilcoxon signed-rank test for dependent variables. Test statistic and p-value will be presented for each comparison. Tests resulting in p-values less than or equal to 0.05 will be reported as "statistically significant".

3. Change of the patient's clinical conditions of vestibular vertigo assessed with SVVSLCRE from baseline to Visit 2 will be presented with median, 1st quartile and 3rd quartile. Comparison between baseline and Visit 2 will be made using Wilcoxon signed-rank test for dependent variables. Test statistic and p-value will be presented for each comparison. Tests resulting in p-values less than or equal to 0.05 will be reported as "statistically significant".

4. Change of vestibular vertigo attacks frequency in number of monthly attacks from baseline to the end of observational treatment period will be summarized with number of patients, mean, standard deviation, median, 1st quartile, 3rd quartile, minimum and maximum values. Descriptive statistics will be also presented for each measure at baseline and the end of observational treatment period. Comparisons of the measures between baseline and the end of observational treatment period will be performed using the two-tailed paired t-test for dependent samples (if the data have approximate normal distribution) or a Wilcoxon signed-rank test for dependent variables (a nonparametric test). Test statistic and p-value will be presented for each comparison. Tests resulting in p-values less than or equal to 0.05 will be reported as "statistically significant".

5. Change of vestibular vertigo attacks frequency in number of monthly attacks from baseline to Visit 2 will be summarized with number of patients, mean, standard deviation, median, 1st quartile, 3rd quartile, minimum and maximum values. Descriptive statistics will be also presented for each measure at baseline and the end of observational treatment period. Comparisons of the measures between baseline and the Visit 2 will be performed using the two-tailed paired t-test for dependent samples (if the data have approximate normal distribution) or a Wilcoxon signed-rank test for dependent variables (a nonparametric test). Test statistic and p-value will be presented for each comparison. Tests resulting in p-values less than or equal to 0.05 will be reported as "statistically significant".

6. Overall clinical response assessed by physicians based on a four-point scale will be presented with number and proportion of patients in each category at the end of observational treatment period for patients who completed betahistine treatment over a period less or equal 30 days and at Visit 2 and 3 for patients who completed betahistine treatment over a period more than 30 days but less or equal 60 days.

7. Overall clinical response assessed by patients based on a four-point scale will be presented with number and proportion of subjects in each category at the end of observational treatment period for patients who completed betahistine treatment over a period less or equal 30 days and at Visit 2 and 3 for patients who completed betahistine treatment over a period more than 30 days but less or equal 60 days.

8. Clinical response as improvement of vertigo associated symptoms (tinnitus, hearing loss, nausea, vomiting, faintness and headache) and evaluated by physicians based on a four-point scale will be presented with number and proportion of patients in each category at the end of observational treatment period for patients who completed betahistine treatment over a period less or equal 30 days and at Visit 2 and 3 for patients who completed betahistine treatment over a period more than 30 days but less or equal 60 days.

9. Clinical response as improvement of vertigo associated symptoms (tinnitus, hearing loss, nausea, vomiting, faintness and headache) and evaluated by patients based on a four-point scale will be presented with number and proportion of subjects in each category at the end of observational treatment period for patients who completed betahistine treatment over a period less or equal 30 days and at Visit 2 and 3 for patients who completed betahistine treatment over a period more than 30 days but less or equal 60 days.

10. Change of vestibular vertigo attacks frequency in number of monthly attacks from the end of observational treatment period to 1st month of follow-up and from the end of observational treatment period to 2nd month of follow-up will be presented for patients who completed betahistine treatment over a period less or equal 30 days and for patients who completed betahistine treatment over a period more than 30 days but less or equal 60 days and will be summarized with number of patients, mean, standard deviation, median, 1st quartile, 3rd quartile, minimum and maximum values. Descriptive statistics will be

also presented for each measure at the end of observational treatment period, 1st month of follow-up and 2nd month of follow-up. Comparisons of the measures between the end of observational treatment period and 1st month of follow-up and between the end observational treatment period and 2nd month of follow-up will be performed using the two-tailed paired t-test for dependent samples (if the data have approximate normal distribution) or a Wilcoxon signed-rank test for dependent variables (a nonparametric test). Test statistic and p-value will be presented for each comparison. Tests resulting in p-values less than or equal to 0.05 will be reported as "statistically significant".

11. Specific underling ICD-10 diagnosis account for Vestibular Vertigo will be summarized with number and proportion of patients in each category.

Other Relevant Topics of Planned Data Analysis

Data analysis will include additional exploratory objectives for which detailed statistical methods will be indicated in separated document - Statistical Analysis Plan. The following exploratory objectives will be under investigation:

1. To assess effectiveness of Betaserc® and the course of vestibular vertigo in post-treatment period in subgroups of patients with different underling diagnosis according to ICD-10.

2. To describe concomitant medication for vestibular vertigo during program period according to underling ICD-10 diagnosis.

3. To describe effectiveness Betaserc® and the course of vestibular vertigo in post-treatment period in the subgroups of sex (males and females) and countries (Russia and Ukraine).

Also, the number and percent of subjects experiencing serious adverse events (SAEs (including deaths) and adverse events leading to discontinuation of program drug will be

tabulated according to the primary MedDRA system organ class (SOC) and MedDRA preferred term.

Sample size considerations

Based on evaluation of literature data that was published by Gananca et al. in Acta Oto-Laryngologica, 2009 [17], proportion of patients with Meniere's Disease achieved control of vertigo (clinical conditions levels 4 and 3) after 12 weeks of treatment with betahistine dihydrochloride tablets constituted 88%. Benecke at al. [18] found that overall efficacy of betahistine in routine clinical practice over 3 months therapy was excellent by 36.6% of 1753 patients with recorded data (n = 641) and good by 49.6% of patients (n = 870), total 86.2 %. The investigators' impression of the treatment was excellent for 38.6% of patients (n = 675 of 1747) and good for 50.5% of patients (n = 883), total 89.1%.

For this program, it is assumed that at least 80% of the total study sample will achieve clinical response after 60 days of therapy with betahistine dihydrochloride tablets. When the sample size without a consideration of dropout is 246, a normal approximation two-sided 95% confidence interval for a single proportion using normal distribution will extend 5,0% (i.e. half width of the 95% confidence interval) from the observed proportion for an expected proportion of 80% [19; 20]. Considering an approximate dropout rate about 20%, total study population (received at least one dose of betahistine dihydrochloride (Betaserc®) tablets and have at least one post-baseline assessment) will constitute 308 patients.

Overall, the best consideration for total number of enrolled patients with vestibular vertigo in the program has to amount 310.

12.0 Final Report and Publications

At the end of the program, a program report will be written in collaboration with the principal investigator(s) (Study Report will be issued within 10 months of Last Patient

Last Visit). This report will contain a description of the objectives of the PMOS, the methodology of the program and its results and conclusions. The completed case report forms and the program report must be treated as the confidential property of Abbott Laboratories and may not be released to unauthorized people in any form (publications or presentations) without express written approval from Abbott Laboratories. The results of this PMOS may be published by Abbott Laboratories or by any one of the participating investigators after written approval of Abbott Laboratories.

13.0 References

1. Морозова С.В., Зайцева О.В., Налетова Н.А. Головокружение как медико-социальная проблема. // РМЖ. - 2002. - № 4. – с.164.
2. Oosterland W.J. Betahistine dihydrochloride in the treatment of vertigo of peripheral vestibular origin. A double-blind placebocontrolled study. J Laryngol Otol 1984; 98:37–41.
3. Yardley L, Owen N, Nazareth I, Luxon L. Prevalence and presentation of dizziness in a general practice community sample of working age people. *Br J Gen Pract.* 1998; 48(429):1131–1135.
4. Colledge N.R., Barr–Hamilton R.M. Evaluation of investigations to diagnose the cause of dizziness in elderly people: a community based controlled study. BMJ. 1996; 313:788 – 92.
5. Claussen C.F. Development of Clinical Neurootological Network Diagnostics During the Last Three Decades. Int Tinnitus J. 2000; 6(2):77.
6. Neuhauser HK Epidemiology of vestibular vertigo. Neurology 2005; 65:898-904.
7. Zamergrad M.V .Vestibular vertigo Neurology, neuropsychiatry, psychosomatics, № 1, 2009, 14-18.
8. Алексеева Н.С. // Материалы симпозиума "Головокружение: современные подходы к решению проблемы" 8–го съезда неврологов России. – М. – 2001. – с. 2 – 5.
9. Благовещенская Н.С. Отоневрологические симптомы и синдромы. – М.: Медицина. – 1990. – 432 с.
10. Thompson T L, Amedee R "Vertigo: A review of common peripheral and central vestibular disorders" The Ochsner Journal 2009, 9: 20-26.
11. Brandt T. Vertigo. Its Multicensory Syndromes. London: Springer, 2000, p. 503.
12. Brandt T, Dieterich M. Vertigo and Dizziness: common complains. London: Springer, 2004; p 208.
13. Debasish Burman1, Saileswar Goswami, Pallab Kumar Majumdar; A Study on Peripheral Vertigo in a Kolkata Based Hospital. Indian Journal of Otolaryngology and Head and Neck Surgery Vol. 54 No. 2, April - June 2002.
14. Alexander G. Reeves, M.D. Disorders of the Nervous System, 2008 Chapter 14.

15. Lacour M, Sterkers O. Histamine and betahistine in the treatment of vertigo. Elucidation of mechanisms of action. CNS Drugs 2001; 15: 853-70.

16. Strupp M, Hupert D, Frenzel C, Wagner J, Hahn A, Jahn K, Zingler VC, Mansmann U, Brandt T. Long-term prophylactic treatment of attacks of vertigo in Menière's disease--comparison of a high with a low dosage of betahistine in an open trial. Acta Otolaryngol. 2008; 128(5):520-4.

17. Ganança MM, Caovilla HH, Ganança FF. Comparable efficacy and tolerability between twice daily and three times daily betahistine for Meniere's disease. Acta Otolaryngol. 2009; 129(5):487-92.

18. Benecke H. et al. Effects of Betahistine on Patient-Reported Outcomes in Routine Practice in Patients with Vestibular Vertigo and Appraisal of Tolerability: Experience in the OSVaLD Study. International Tinnitus Journal. 2010; 16(1):14-24.

19. Fleiss, J. L., Levin, B., Paik, M.C. 2003. Statistical Methods for Rates and Proportions. Third Edition. John Wiley & Sons. New York.

20. Newcombe, R. G. 1998. 'Two-Sided Confidence Intervals for the Single Proportion: Comparison of Seven Methods.' Statistics in Medicine, 17, pp. 857-872.

21. Neuhauser HK, von Brevern M, Radtke A, Lezius F, Feldmann M, Ziese T, Lempert T. Epidemiology of vestibular vertigo: a neurotologic survey of the general population. Neurology. 2005; 65(6):898-904.

22. Sloane P.D. Dizziness in primary care: results from the National Ambulatory Medical Care Survey. J Fam Pract 1989; 29:33-38.

23. Redon C, Lopez C, Bernard-Demanze L, Dumitrescu M, Magnan J, Lacour M, Borel L. Betahistine treatment improves the recovery of static symptoms in patients with unilateral vestibular loss. J Clin Pharmacol. 2011; 51(4):538-48.

24. Lacour M, Sterkers O. Histamine and betahistine in the treatment of vertigo: elucidation of mechanisms of action. CNS Drugs. 2001; 15(11):853-70.

14.0 Appendix: Protocol Amendment 1: List of Changes

<u>Specific Changes</u> Amendment 1, dated 10 December 2012

1.1 Protocol Amendment 1: Summary of Changes

Section was added to describe changes under Amendment 1.

Section 7.5 Program Conduct

Figure 1: Overall Investigational Plan of Study previously read:

Figure 2: Overall Investigational Plan of Study

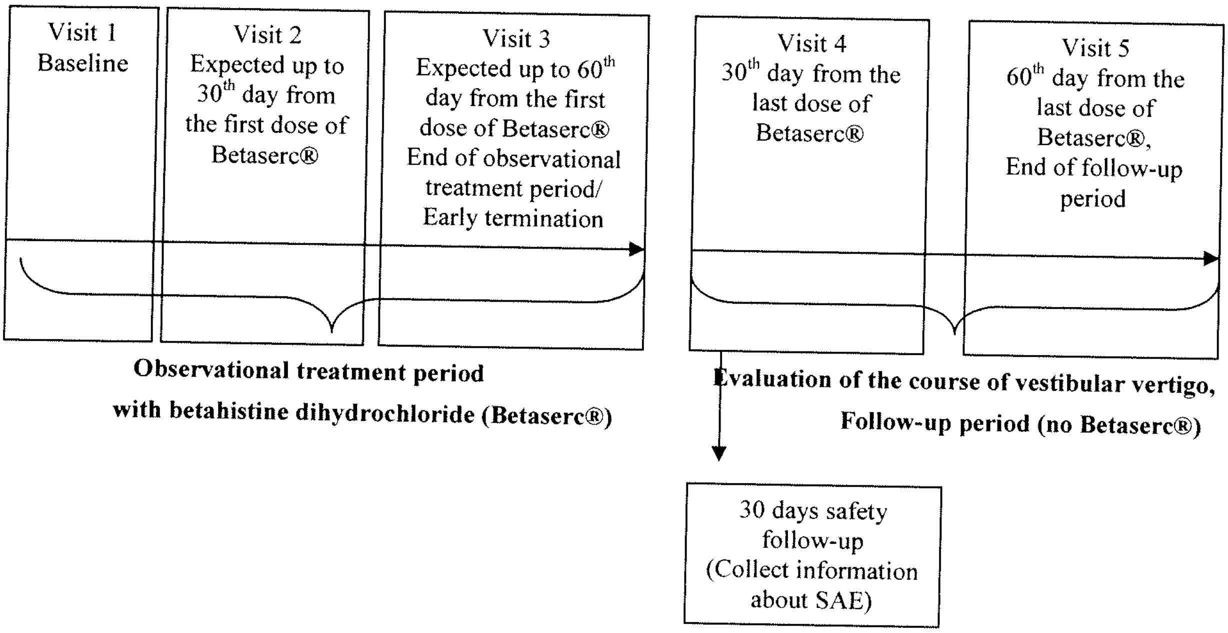

Has been changed to read:

Figure 2: Overall Investigational Plan of Study

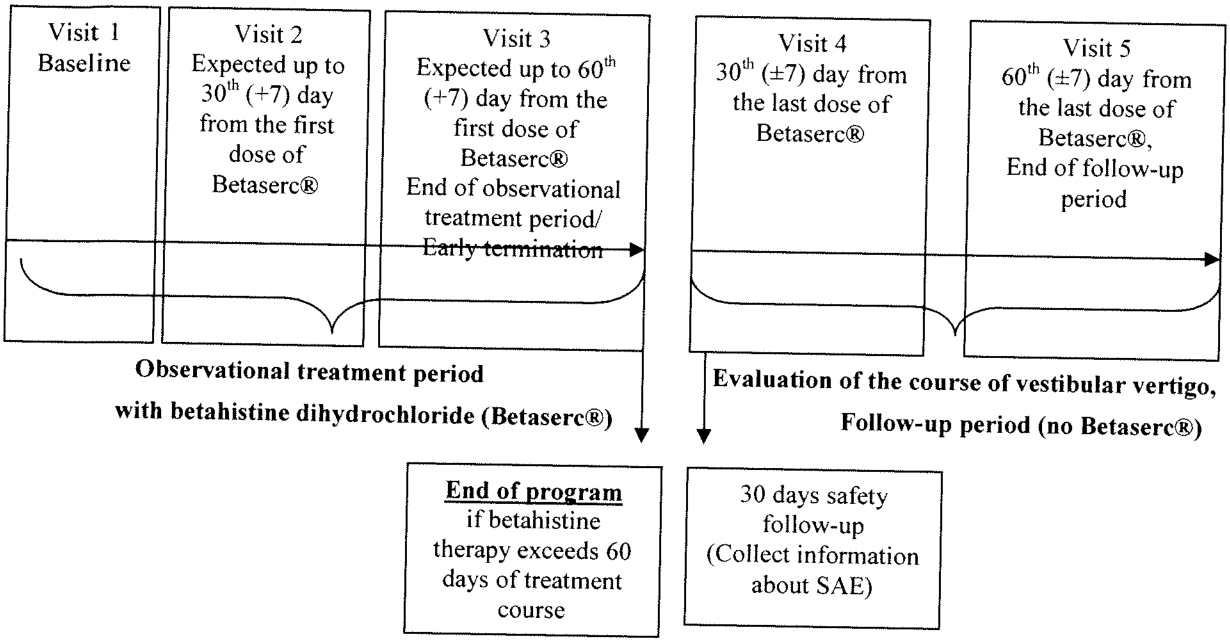

Reason for changes: figure clarification to be in line with the program conduct and figure number according to the correct appearance in the text.

Table 1 Program Activities previously read:

Weeks of Treatment		Observational treatment period[1]		Evaluation of the course of vestibular vertigo in follow-up period	
	Visit 1	Visit 2	Visit 3	Visit 4	Visit 5
		Expected up to 30th day from the first dose of Betaserc®[2]	Expected up to 60th day from the first dose of Betaserc®[2]	30th (±7) day from the last dose of Betaserc®	60th (±7) day from the last dose of Betaserc®
	Screening/ Baseline		End of observational treatment period[3,4]/Early termination		End of follow-up period
Patient Authorization (Consent) for Use/Disclosure of Data	X				
Inclusion / exclusion criteria	X				
Register date of start of betahistine dihydrochloride (Betaserc®) treatment	X				
Demographics[5]	X				
Medical history	X				
Concomitant medication	X	X	X	X	X
Physical Exam (including vitals[6])	X				
Specific underlying ICD-10 diagnosis account for Vestibular Vertigo	X				
Number of monthly vertigo attacks	X	X	X	X	X
Scale for Vestibular Vertigo Severity Level and Clinical Response Evaluation[7]	X	X	X		
Overall clinical response and clinical response as improvement of vestibular vertigo associated symptoms based on four-point scales[8]		X	X		
SAE monitoring[9]	X	X	X	X	

Has been changed to read:

Table 2 Program Activities

Weeks of Treatment		Observational treatment period[1]		Evaluation of the course of vestibular vertigo in follow-up period	
	Visit 1	Visit 2	Visit 3	Visit 4	Visit 5
		Expected up to 30th day from the first dose of Betaserc®[2]	Expected up to 60th day from the first dose of Betaserc®[2]	30th (±7) day from the last dose of Betaserc®	60th (±7) day from the last dose of Betaserc®
	Screening/ Baseline		End of observational treatment period[3,4]/Early termination		End of follow-up period
Patient Authorization (Consent) for Use/Disclosure of Data	X				
Inclusion / exclusion criteria	X				
Register date of start of betahistine dihydrochloride (Betaserc®) treatment	X				
Demographics[5]	X				
Medical history	X				
Concomitant medication	X	X	X	X	X
Physical Exam (including vitals[6])	X				
Specific underlying ICD-10 diagnosis account for Vestibular Vertigo	X				
Number of monthly vertigo attacks	X	X	X	X	X
Scale for Vestibular Vertigo Severity Level and Clinical Response Evaluation[7]	X	X	X		
Overall clinical response and clinical response as improvement of vestibular vertigo associated symptoms based on four-point scales[8]		X	X		
SAE monitoring[9]	X	X	X	X	

Reason for changes: table number according to the correct appearance in the text.

Section 7.5.2 Description of Activities

Visit 4 and Visit 5 activities previously read:

Visit 4, 30th (±7) day from the last dose of Betaserc®:

- Register concomitant medication
- Register of the number of vertigo attacks for the previous month
- Evaluate patient's clinical conditions based on a follow-up four level scale
- SAE monitoring

Visit 5, 60th (±7) day from the last dose of Betaserc®:

- Register concomitant medication
- Register of the number of vertigo attacks for the previous month
- Evaluate patient's clinical conditions based on a follow-up four level scale

Has been changed to read:

Visit 4, 30th (±7) day from the last dose of Betaserc®:

- Register concomitant medication
- Register of the number of vertigo attacks for the previous month
- SAE monitoring

Visit 5, 60th (±7) day from the last dose of Betaserc®:

- Register concomitant medication
- Register of the number of vertigo attacks for the previous month

Reason for changes: Removal of «Evaluate patient's clinical conditions based on a follow-up four level scale» item to be in line with planned activities according to Table 2 Program Activities.

Section 9.0 Ethics and Quality previously read:

Ukraine: the protocol and relevant study documents will be submitted for review and approval to Ethics Committee of Ministry of Health of Ukraine by Abbott. It is the responsibility of each investigator to obtain study approval of an Independent Local Ethics Committee before initiating the study, if applicable.

Has been changed to read:

Ukraine: the protocol and relevant study documents will be submitted for review and approval to State Expert Center of Ministry of Health of Ukraine by Abbott. It is the responsibility of each investigator to obtain study approval (if applicable) of local IEC before initiating the study.

Reason for changes: changes in local regulation.

Abbott Laboratories

Post-Marketing Observational Program Protocol (P13-972)

Amendment 1

Post-Marketing Observational Program of Betaserc® (Betahistine dihydrochloride) to Evaluate Effectiveness in Patients with Vestibular Vertigo in Routine Practice (VIRTUOSO)

Approved by:

Alexander Vladykin [signature]
Protocol Author - Alexander Vladykin/Senior Clinical Research Associate — 12.12.2012 Date

Marina Matrosova [signature]
Study-Designated Physician - Marina Matrosova/Medical Advisor — 12.12.12. Date

Ruslan Nafeiev [signature]
Additional Reviewer - Ruslan Nafieiev/CIS Medical Adviser — 12.12.12 Date

Jos Nauta [signature]
Statistics - Jos Nauta / Statictician — 12-12-12 Date

K. Giezeman [signature]
Project Director - Katinka Giezeman / Senior Medical Director, EPD — 12-12-12 Date
