# Supplementary material for: Effectiveness of betahistine (48 mg/day) in patients with vestibular vertigo during routine practice: The VIRTUOSO study
Source: PLoS One. 2017 Mar 30;12(3):e0174114. doi: 10.1371/journal.pone.0174114 (PMC5373561; doi:10.1371/journal.pone.0174114)
Supplement: S4 Fig — (PDF) [file pone.0174114.s008.pdf]

1.0 Титульная страница

Эбботт Лэбораториз

Постмаркетинговая наблюдательная программа Протокол (P13-972)

Поправка 1

Постмаркетинговая наблюдательная программа по оценке эффектов препарата Бетасерк® (бетагистина дигидрохлорид) у пациентов с вестибулярным головокружением в рутинной клинической практике (VIRTUOSO)

Название препарата:	Бетагистина дигидрохлорид (Бетасерк®)	
Тип исследования:	Постмаркетинговая наблюдательная программа	
Дата:	10 декабря 2012 года	
Спонсор:	**Эбботт Лэбораториз**<br>БЦ "Метрополис"<br>Ленинградское шоссе, д. 16А, строение 1,<br>Москва 125171 Российская Федерация	Тел.: +7 495 258 4280<br>Факс: +7 495 258 4281
	**Эбботт Украина**<br>ул. Жилянская, д. 110, 2й этаж<br>Киев, 01032 Украина	Тел.: +38 044 498 60 80<br>Факс: +38 044 498 60 81

1.1 Обзор изменений

Настоящая поправка к протоколу включает ряд технических и административных изменений.

Перечень изменений, содержащихся в данной поправке к протоколу, приведен в Приложении.

2.0 Содержание

1.0 Титульная страница 1
1.1 Обзор изменений 2
2.0 Содержание 3
3.0 Список сокращений 5
4.0 Введение 6
5.0 Обоснование 8
6.0 Цели программы 9
6.1 Первичная цель 9
6.2 Вторичные цели 9
6.3 Первичная конечная точка 10
6.4 Вторичные конечные точки 12
7.0 План программы 15
7.1 Отбор исследуемой популяции 15
7.1.1 Критерии включения 16
7.1.2 Критерии исключения 17
7.2 Планируемое количество пациентов 18
7.3 Критерии отбора исследователей 18
7.4 Продолжительность наблюдательной программы 18
7.5 Проведение наблюдательной программы 19
7.5.1 Поставки препарата 23
7.5.2 Процедуры наблюдательной программы 23
8.0 Нежелательные явления 26
8.1 Определение нежелательного явления и категорий серьезного нежелательного явления 26
8.2 Степень тяжести 28
8.3 Связь с фармацевтическим препаратом 28
8.4 Период регистрации серьезных нежелательных явлений 29
8.5 Сообщения о серьезных нежелательных явлениях 29
8.6 Сообщение о беременности 30
9.0 Этика и качество 30
10.0 Индивидуальная регистрационная карта 32

11.0 План анализа данных 32
12.0 Итоговый отчет и публикации 41
13.0 Список литературы 42
14.0 Приложение: Поправка к протоколу 1: Перечень изменений 45

3.0 Список сокращений

ЛОР	Оториноларингология
МКБ-10	Международная классификация болезней 10-го пересмотра
НЭК	Независимый этический комитет
НЯ	Нежелательное явление
СНЯ	Серьезное нежелательное явление
эИРК	Электронная индивидуальная регистрационная карта
ЭСД	Электронный сбор данных
MedDRA	Медицинский словарь терминов MedDRA

4.0 Введение

Головокружение − один из наиболее частых симптомов, встречающихся в клинической практике, который может являться проявлением более 80 заболеваний. На головокружение предъявляют жалобы приблизительно 5% пациентов врачей общей практики [1] и 10% пациентов, обращающихся к отоларингологам [2]. Согласно данным Yardley L., полученным в ходе опроса 20 тысяч человек в возрасте от 18 до 64 лет, более чем у 20% субъектов головокружение наблюдалось в течение месяца до опроса, а у 30% в течение последних 5 лет [3]. Из 1000 опрошенных лиц старше 60 лет, 30% отмечали головокружение в течение жизни [4]. Приблизительно каждый третий больной, посещающий отоларинголога, предъявляет жалобы на головокружение [5].

Выделяют два вида головокружения: вестибулярное и невестибулярное. Вестибулярное головокружение, которое часто определяют как иллюзию перемещения в пространстве, является результатом нарушений вестибулярного аппарата [6]. Вестибулярное головокружение может быть центральным − при поражении структур центральной нервной системы (вестибулярные ядра ствола мозга, вестибулярные центры и вестибулярные проводящие пути головного мозга) или периферическим (поражение вестибулярного нерва или лабиринта). Вестибулярное головокружение является распространенной проблемой, встречающейся у более 5% взрослых людей [6; 7]. К развитию вестибулярного головокружения предрасполагают следующие факторы: женский пол, возраст, низкий уровень образования, сопутствующие заболевания (включая, депрессию, звон в ушах, некоторые сердечно-сосудистые заболевания, например, дислипидемия и гипертоническая болезнь) и другие [21].

Вестибулярное головокружение классифицируют по уровню поражения вестибулярного анализатора (центральная или периферическая часть вестибулярного анализатора) [14]. Головокружение, связанное с поражением центров равновесия головного мозга, обычно проявляется менее интенсивно и

сопровождается неврологическими симптомами, такими как нарушения речи, двоение в глазах и патологический нистагм [7; 11; 12, 13]. Повреждения головного мозга могут вызывать ощущение потери равновесия.

Целый ряд заболеваний центральной нервной системы может сопровождаться головокружением: мигрень, латеральный синдром продолговатого мозга, рассеянный склероз [8; 9]. Вестибулярное головокружение может сопровождаться различными формами нистагма, включая спонтанный горизонтальный оптокинетический нистагм.

Периферическое вестибулярное головокружение характеризуется эпизодическим или постоянным ощущением качания [10]. Болезнь Меньера часто вызывает эпизоды головокружения, которые развиваются в течение минут, продолжаются несколько часов, с постепенным разрешением на протяжении последующих нескольких часов. Вестибулярный нейронит и лабиринтит обычно начинаются остро, в течение нескольких часов развивается головокружение, которое, по прошествии острой фазы, постепенно разрешается на протяжении нескольких дней. Травмы или ишемические повреждения лабиринта вызывают острое появление симптомов, которые медленно разрешаются после острой фазы в течение нескольких дней или недель, часто с остаточными явлениями, длящимися несколько месяцев [22].

Лечение вестибулярного головокружения может включать различные методы в зависимости от характера основного заболевания. Некоторые виды головокружения могут потребовать дополнительной терапии и консультаций узких специалистов.

Бетагистин

Бетагистин был впервые зарегистрирован в 1968 году в Канаде, и к настоящему времени одобрен более чем в 115 странах для лечения болезни Меньера и симптоматического лечения головокружения. Увеличение количества гистамина, высвобождающегося из гистаминовых нервных окончаний, стимулирует H1-

рецепторы, усиливая, в результате, прямое стимулирующее действие бетагистина на эти рецепторы. Данный механизм объясняет известное выраженное сосудорасширяющее действие бетагистина в структурах внутреннего уха [15]. Уменьшение количества приступов головокружения при лечении бетагистином обусловлено увеличением тока крови во внутреннем ухе, что снижает повышенное эндолимфатическое давление во внутреннем ухе, восстанавливая баланс между продукцией и обратным всасыванием эндолимфы [16]. Кроме того, в различных исследованиях на животных показано, что бетагистин способен улучшать вестибулярную компенсацию, проявляя прямое гистаминергическое действие; препарат ускоряет высвобождение и метаболизм гистамина, блокируя пресинаптические гистаминовые рецепторы Н3 (в вестибулярных и туберомамиллярном ядрах) [15].

В клинической практике в России и на Украине, бетагистина дигидрохлорид назначается перорально в дозах по 24-48 мг/сутки в качестве монотерапии или в комбинации с другими препаратами, когда применяемая схема лечения головокружения характеризуется недостаточной эффективностью или переносимостью.

5.0 Обоснование

Целью настоящей международной постмаркетинговой наблюдательной программы является оценка эффектов таблеток бетагистина дигидрохлорида (Бетасерк®) и изучение течения вестибулярного головокружения после завершения курса приема препарата в ходе наблюдения за популяцией амбулаторных пациентов из России и Украины, страдающих вестибулярным головокружением и получающих лечение в рамках рутинной клинической практики. Специальной целью данной программы является изучение назначения бетагистина дигидрохлорида в максимальной рекомендованной суточной дозе (48 мг) в соответствии с утвержденной

инструкцией по медицинскому применению препарата в рамках сложившейся клинической практики.

Хорошо известно, что 2−3 месячный курс лечения бетагистина дигидрохлоридом может ускорять или облегчать вестибулярную компенсацию у пациентов с вестибулярными нарушениями [23, 24]; поэтому целью данной программы также является оценка течения вестибулярного головокружения после отмены препарата Бетасерк®. Период последующего наблюдения продолжительностью два месяца выбран в связи с тем, что рядом авторов такой промежуток времени считается достаточным для оценки рецидива развития симптомов большинства неврологических и отоларингологических заболеваний, сопровождающихся головокружением (неопубликованные данные российских и украинских неврологов и отоларингологов).

Поисковый анализ результатов настоящей программы, полученных благодаря участию пациентов обеих стран, может предоставить дополнительные данные об особенностях вестибулярного головокружения в широкой популяции пациентов с различными основными диагнозами, культурными и национальными особенностями.

6.0 Цели программы

6.1 Первичная цель

1. Оценка эффектов применения бетагистина дигидрохлорида в дозе 48 мг/сутки в форме таблеток в рутинной клинической практике у амбулаторных пациентов с вестибулярным головокружением.

6.2 Вторичные цели

1. Оценить общий клинический ответ, определяемый как регресс связанных с головокружением симптомов в течение периода терапии.

2. Оценить течение вестибулярного головокружения на протяжении 2-месячного периода последующего наблюдения после завершения курса приема препарата.
3. Описать согласно МКБ-10 основной диагноз, сопровождающийся головокружением.

6.3 Первичная конечная точка

1. Количество и доля пациентов с клиническим ответом на момент завершения терапии в ходе наблюдения в рамках программы, при оценке по Шкале тяжести вестибулярного головокружения и клинического ответа (SVVSLCRE) (Рис. 1 и Таб. 1), в общей исследуемой популяции.

Пациенты будут оценивать тяжесть головокружения по шкале SVVSLCRE при включении в программу и на момент завершения периода терапии в ходе наблюдения в рамках исследования.

Рисунок 1. Шкала оценки тяжести вестибулярного головокружения и клинического ответа (SVVSLCRE).

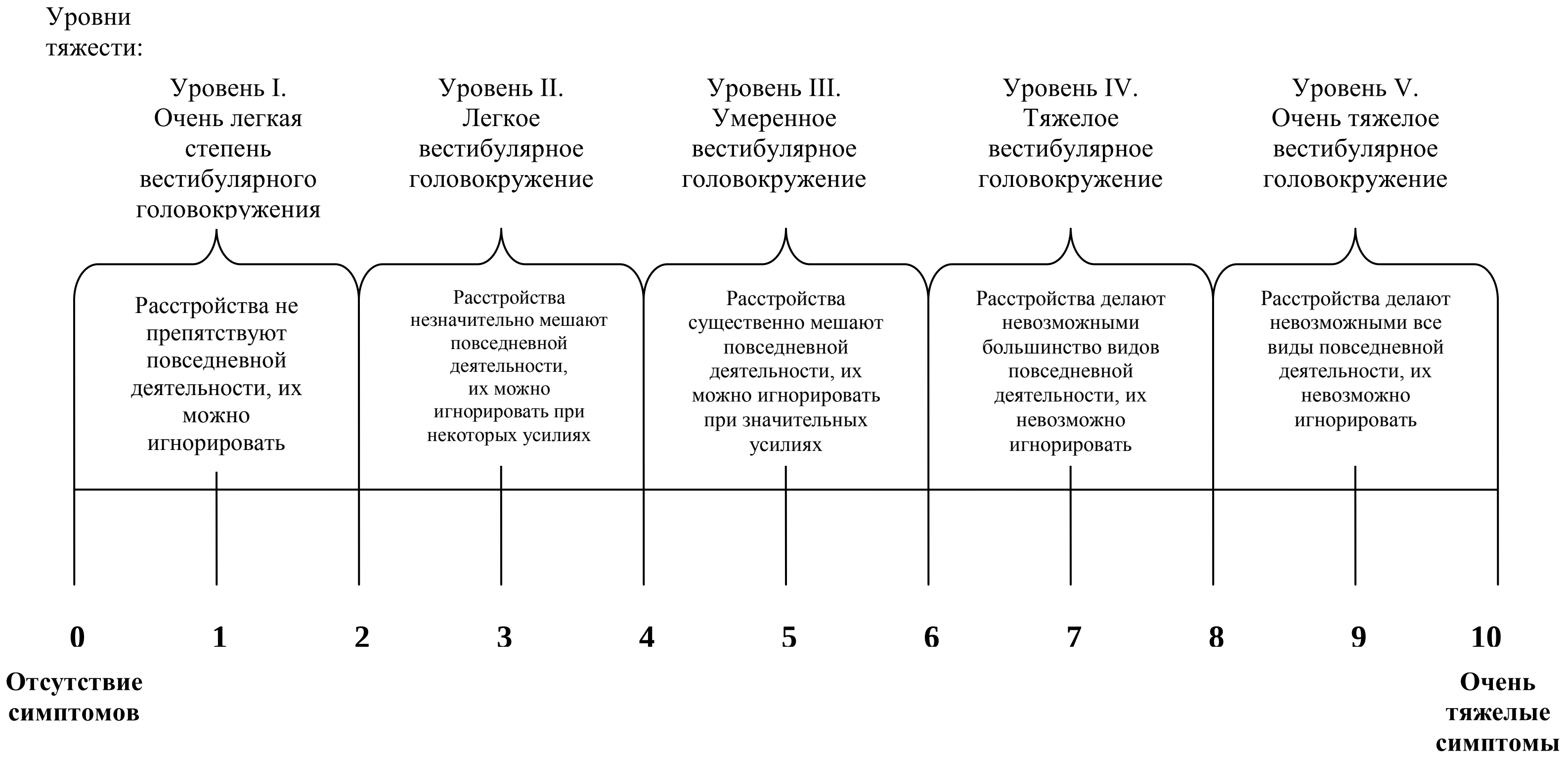

Таблица 1. Клинический ответ определяли следующим образом:

Изменения в клиническом течении вестибулярного головокружения	*Изменения по шкале SVVSLCRE на момент завершения терапии в ходе наблюдения в рамках программы по сравнению с исходным значением при включении в программу*
Ухудшение	Все прочие изменения
Отсутствие изменений	с V на V; с IV на IV; с III на III; с I на I
Незначительное улучшение	с V на IV; с IV на III; с III на II; с II на I
Значительное улучшение	с V на III; с IV на II; с III на I
Выраженное улучшение	с V на II; с IV на I
Очень выраженное улучшение	с V на I

6.4 Вторичные конечные точки

1. Количество и доля пациентов в каждой категории по шкале SVVSLCRE при включении в программу и на момент завершения терапии в ходе наблюдения в рамках программы среди пациентов, получавших лечение бетагистина дигидрохлоридом в течение ≤30 дней, и среди пациентов, получавших бетагистина дигидрохлорид >30 дней, но ≤60 дней.

2. Изменение в степени тяжести вестибулярного головокружения по шкале SVVSLCRE на момент завершения терапии в ходе наблюдения в рамках программы по сравнению с визитом включения в программу среди пациентов, получавших лечение бетагистина дигидрохлоридом в течение ≤30 дней, и среди пациентов, получавших бетагистина дигидрохлорид >30 дней, но ≤60 дней.

3. Изменение в степени тяжести вестибулярного головокружения по шкале SVVSLCRE на Визите 2 по сравнению с визитом включения в программу у пациентов, получавших бетагистина дигидрохлорид >30 дней, но ≤60 дней.

4. Изменение частоты эпизодов вестибулярного головокружения на момент завершения терапии в ходе наблюдения в рамках программы по сравнению с визитом включения в программу среди пациентов, получавших лечение бетагистина дигидрохлоридом в течение ≤30 дней, и среди пациентов, получавших бетагистина дигидрохлорид >30 дней, но ≤60 дней.

5. Изменение частоты эпизодов вестибулярного головокружения на Визите 2 по сравнению с визитом включения в программу у пациентов, получавших бетагистина дигидрохлорид >30 дней, но ≤60 дней.

6. Общий клинический ответ по оценке врача, определяемый по 4-бальной шкале, где 4 = выраженное улучшение, 3 = значительное улучшение, 2 = незначительное улучшение и 1 = ухудшение. Доля пациентов в каждой категории на момент завершения терапии в ходе наблюдения в рамках программы будет определяться для пациентов, получавших лечение бетагистина дигидрохлоридом в течение ≤30 дней, и среди пациентов, получавших бетагистина дигидрохлорид >30 дней, но ≤60 дней. Доля пациентов в каждой категории на Визите 2 будет определяться для пациентов, получавших бетагистина дигидрохлорид >30 дней, но ≤60 дней.

7. Общий клинический ответ по оценке пациента, определяемый по 4-бальной шкале, где 4 = выраженное улучшение, 3 = значительное улучшение, 2 = незначительное улучшение и 1 = ухудшение. Доля пациентов в каждой категории на момент завершения терапии в ходе наблюдения в рамках программы будет определяться для пациентов, получавших лечение бетагистина дигидрохлоридом в течение ≤30 дней, и среди пациентов, получавших бетагистина дигидрохлорид >30 дней, но ≤60 дней. Доля пациентов в каждой категории на Визите 2 будет определяться для пациентов, получавших бетагистина дигидрохлорид >30 дней, но ≤60 дней.

8. Регресс связанных с головокружением симптомов (звон в ушах, нарушение слуха, тошнота, рвота, слабость и головная боль) по мнению врача, оцениваемые по 4-

бальной шкале, где 4 = выраженное улучшение, 3 = значительное улучшение, 2 = незначительное улучшение и 1 = ухудшение. Доля пациентов в каждой категории на момент завершения терапии в ходе наблюдения в рамках программы будет определяться для пациентов, получавших лечение бетагистина дигидрохлоридом в течение ≤30 дней, и среди пациентов, получавших бетагистина дигидрохлорид >30 дней, но ≤60 дней. Доля пациентов в каждой категории на Визите 2 будет определяться для пациентов, получавших бетагистина дигидрохлорид >30 дней, но ≤60 дней.

9. Регресс связанных с головокружением симптомов (звон в ушах, нарушение слуха, тошнота, рвота, слабость и головная боль) по мнению пациента, оцениваемый по 4-бальной шкале, где 4 = выраженное улучшение, 3 = значительное улучшение, 2 = незначительное улучшение и 1 = ухудшение. Доля пациентов в каждой категории на момент завершения терапии в ходе наблюдения в рамках программы будет определяться для пациентов, получавших лечение бетагистина дигидрохлоридом в течение ≤30 дней, и среди пациентов, получавших бетагистина дигидрохлорид >30 дней, но ≤60 дней. Доля пациентов в каждой категории на Визите 2 будет определяться для пациентов, получавших бетагистина дигидрохлорид >30 дней, но ≤60 дней.

10. Изменение частоты эпизодов вестибулярного головокружения в конце первого месяца периода последующего наблюдения по сравнению с завершением периода терапии в ходе наблюдения в рамках программы среди пациентов, получавших лечение бетагистина дигидрохлоридом в течение ≤30 дней, и среди пациентов, получавших бетагистина дигидрохлорид >30 дней, но ≤60 дней.

11. Изменение частоты эпизодов вестибулярного головокружения в конце второго месяца периода последующего наблюдения по сравнению с завершением периода терапии в ходе наблюдения в рамках программы среди пациентов, получавших лечение бетагистина дигидрохлоридом в течение ≤30 дней, и среди пациентов, получавших бетагистина дигидрохлорид >30 дней, но ≤60 дней.

12. Основной неврологический/оториноларингологический диагноз по МКБ-10, сопровождающийся вестибулярным головокружением. Доля пациентов в каждой категории диагнозов на Визите 1 будет рассчитана для общей популяции исследования.

7.0 План программы

Проспективная, многоцентровая, наблюдательная, неинтервенционная, постмаркетинговая программа с участием субъектов одной группы без рандомизации и группы контроля.

7.1 Отбор исследуемой популяции

Настоящая проспективная, многоцентровая, несравнительная, постмаркетинговая наблюдательная программа планируется к проведению с участием пациентов, которым таблетки бетагистина дигидрохлорида (Бетасерк®) были назначены в максимальной рекомендованной суточной дозе (48 мг) в соответствии с утвержденной документацией по препарату (инструкцией по применению/сводной характеристикой лекарственного средства). В течение всего периода проведения программы врач-исследователь может изменить дозу препарата Бетасерк® в соответствии с утвержденной документацией по препарату.

В программу могут быть включены взрослые пациенты, получающие лечение бетагистина дигидрохлоридом в соответствии с утвержденной инструкцией по применению препарата для лечения вестибулярного головокружения. Данная программа не предполагает включение пациентов, требующих, по мнению врача, лечения бетагистином на протяжении более 2 месяцев.

Изучаемая суточная доза препарата 48 мг, может быть разделена на несколько приемов следующим образом:

Таблетки по 8 и 16 мг

Таблетки по 8 мг	Таблетки по 16 мг
2 таблетки<br>3 раза в день	1 таблетка<br>3 раза в день

Таблетки по 24 мг
Суточная доза для взрослых − 48 мг, разделенная на несколько приемов.

Таблетки по 24 мг
1 таблетка<br>2 раза в день

Назначение бетагистина дигидрохлорида пациенту проводится до включения его в наблюдательную программу, в рамках установленной клинической практики. Назначение бетагистина дигидрохлорида (Бетасерк®) не связано с решением включить пациента в данную наблюдательную программу. Никакие дополнительные процедуры обследования (кроме принятых в стандартной клинической практике) в данной программе у пациентов применяться не будут.

До включения в программу пациенты должны предоставить письменное согласие врачу-исследователю на использование и обработку персональных данных.

7.1.1 Критерии включения

1. Мужчины и женщины в возрасте ≥ 18 лет.

2. Пациенты с вестибулярным головокружением известной или неизвестной этиологии, которым врач назначил лечение бетагистина дигидрохлоридом (Бетасерк®) в суточной дозе 48 мг, согласно утвержденной инструкции по применению препарата.

3. Пациенты, предоставившие согласие на использование и обработку персональных данных.

4. Пациенты, которые начали лечение бетагистина дигидрохлоридом (Бетасерк®) не ранее, чем за 5 дней до подписания согласия на использование и обработку персональных данных.

7.1.2 Критерии исключения

1. Пациенты с заболеваниями, которые, по мнению врача-исследователя, делают невозможным включение пациента в данную программу.

2. Известные противопоказания к применению бетагистина дигидрохлорида (Бетасерком®).

3. Пациенты с инфекциями среднего или внутреннего уха.

4. Пациенты с психиатрическими заболеваниями, существенными неврологическими расстройствами или поражением спинного мозга.

5. Пациенты, получающие любые другие препараты для лечения периферического вестибулярного головокружения, например, диуретики, внутриушное введение гентамицина, циннаризин, конкурентный антагонист гистамина, блокирующий H1-гистаминовые рецепторы.

6. Пациенты с оперативными вмешательствами на ухе по поводу вестибулярных расстройств.

7. Беременные, планирующие беременность или кормящие грудью женщины.

8. Неспособность пациента следовать требованиям протокола.

9. Участие в любом другом клиническом исследовании в течение последних 30 дней.

10. Пациенты, получавшие лечение бетагистином в течение 4 недель до предполагаемого начала лечения бетагистином (Бетасерк®) в рамках данной программы.

7.2 Планируемое количество пациентов

В настоящую программу планируется включить 310 пациентов из приблизительно 10-35 центров на территории России и Украины.

7.3 Критерии отбора исследователей

При выборе исследователей для участия в данной программе будут использоваться следующие критерии:

1) Специалисты в области оториноларингологии или неврологии.
2) Возможность надлежащего проведения наблюдательной программы.
3) Доступность амбулаторных пациентов с вестибулярным головокружением.

7.4 Продолжительность наблюдательной программы

Пациенты будут наблюдаться приблизительно в течение 2 месяцев на протяжении периода терапии препаратом Бетасерк® и на протяжении 2 месяцев периода последующего наблюдения для оценки течения вестибулярного головокружения после завершения курса приема препарата. В зависимости от основного диагноза по МКБ-10 и решения врача-исследователя, продолжительность периода лечения может быть менее 2 месяцев или может продолжаться после завершения периода терапии, предусмотренного настоящей программой. Однако только пациенты, получавшие лечение бетагистина дигидрохлоридом (Бетасерк®) в течение 1-2 месяцев будут включены в 2-месячный период последующего наблюдения в рамках настоящей наблюдательной программы.

7.5 Проведение наблюдательной программы

Визиты пациента будут спланированы с учетом графика работы врача-исследователя. Продолжительность срока наблюдения в период проведения терапии 60 дней была выбрана, как достаточная для получения значимых данных по эффектам препарат. Периодичность и время визитов в течение периода последующего наблюдения будут планироваться с учетом обычного графика консультаций лечащего врача.

В целях оптимизации сбора данных рекомендуется планировать три визита в течение 2 месяцев периода терапии и два визита в течение 2 месяцев периода последующего наблюдения:

I. Период терапии в ходе наблюдения в рамках программы

Визит 1: Получение исходных данных/Начало терапии.

Визит 2: до 30-го дня после приема первой дозы Бетасерк®.

Визит 3: до 60-го дня после приема первой дозы Бетасерк®, завершение периода терапии в ходе наблюдения в рамках программы/преждевременное завершение участия в программе.

II. Оценка течения вестибулярного головокружения после завершения периода терапии в ходе наблюдения в рамках программы, период последующего наблюдения (только для пациентов, получавших бетагистина дигидрохлорид (Бетасерк®) в рамках программы в течение 1-2 месяцев)

Визит 4: 30-й день после приема последней дозы Бетасерк®.

Визит 5: 60-й день после приема последней дозы Бетасерк®, завершение периода последующего наблюдения.

В период последующего наблюдения персонал клинического центра будет контактировать с пациентами лично или по телефону через 30 дней после назначения последней дозы препарата Бетасерк® для выявления возможных СНЯ,

развившихся после завершения периода терапии, а также для получения новой информации о любых СНЯ, развившихся во время лечения.

На Рисунке 2 представлена схема проведения программы, в Таблице 2 приведены основные процедуры наблюдательной программы, проводимые во время визитов.

Рисунок 2: Общий план проведения наблюдательной программы

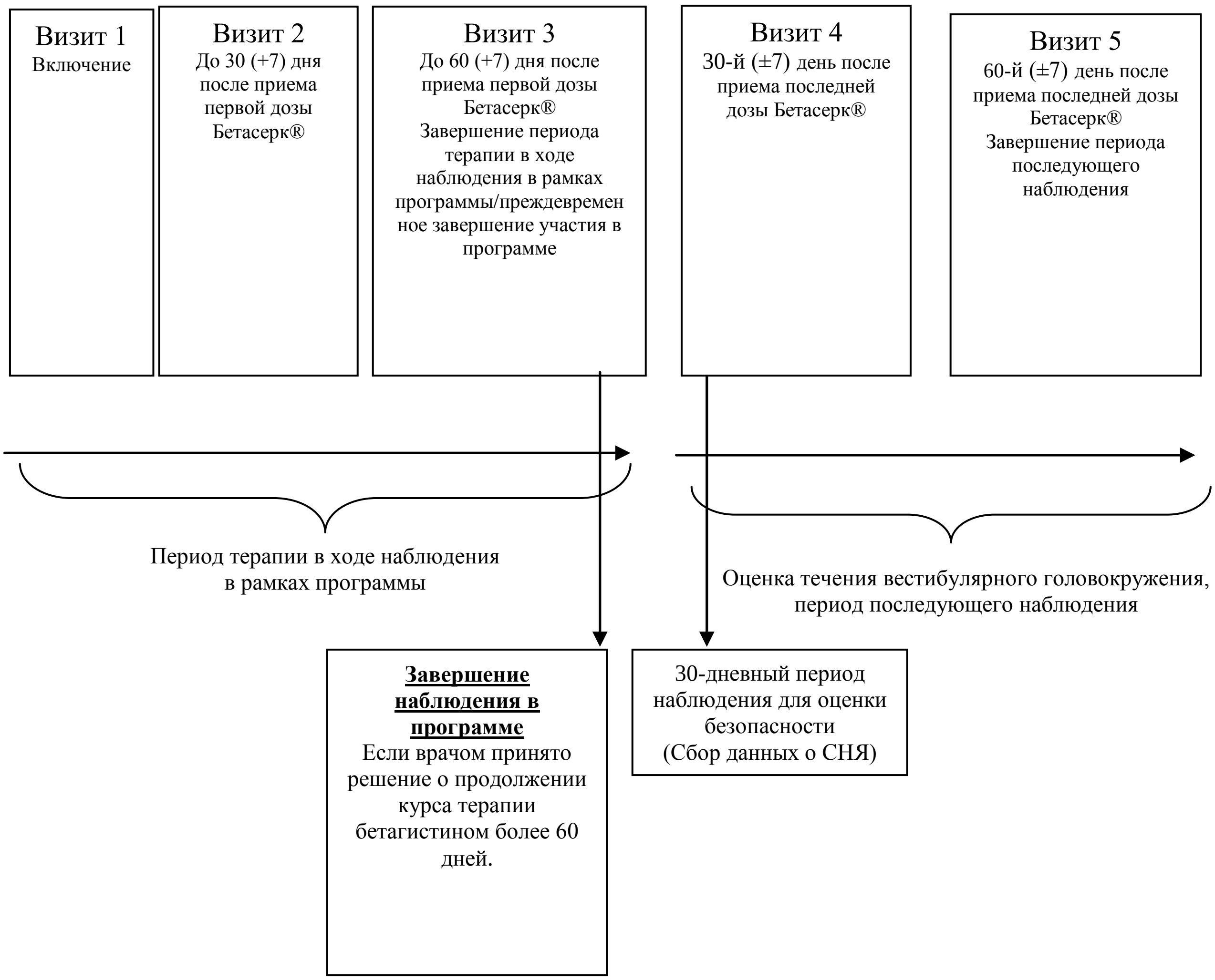

Таблица 2 Процедуры наблюдательной программы

		Период терапии в ходе наблюдения в рамках программы[1]		Оценка течения вестибулярного головокружения в период последующего наблюдения	
	**Визит 1**	**Визит 2**	**Визит 3**	**Визит 4**	**Визит 5**
		В течение 30 дней после приема первой дозы Бетасерк®[2]	В течение 60 дней после приема первой дозы Бетасерк®[2]	30й (±7) день после приема последней дозы Бетасерк®	60й (±7) день после приема последней дозы Бетасерк®
	Скрининг/ Включение, сбор исходных данных		Завершение периода терапии в рамках наблюдения в ходе программы [3,4]/ преждевременное завершение участия в программе		Завершение периода последующего наблюдения
Согласие на использование и обработку персональных данных	X				
Оценка критериев включения / исключения	X				
Регистрация даты начала применения бетагистина дигидрохлорида (Бетасерк®)	X				
Сбор демографических данных[5]	X				
Сбор анамнеза	X				
Регистрация сопутствующих препаратов	X	X	X	X	X
Физикальное обследование (включая оценку основных витальных функций организма[6])	X				
Диагноз по МКБ-10, симптомом которого является вестибулярное головокружение	X				
Количество приступов головокружения в месяц	X	X	X	X	X
Шкала оценки тяжести вестибулярного головокружения и клинического ответа (SVVSLCRE)[7]	X	X	X		
Общий клинический ответ и регресс связанных с вестибулярным головокружением симптомов по 4-бальной шкале[8]		X	X		
Мониторинг серьезных НЯ[9]	X	X	X	X	

[1]В данную программу не предполагается включение пациентов, требующих, по мнению врача, проведение терапии бетагистином на протяжении более 60 дней.

[2] Фактическая дата визита может состояться в течение 7 дней после приема последней дозы бетагистина, в противном случае следует планировать Визит 4.

[3] Совпадает с Визитом 2 у пациентов, получавших бетагистин в течение 30 дней или меньше. В случае, если врач решит продолжить лечение бетагистином более 30 дней, Визит 3 не будет совпадать с Визитом 2.

[4] Для пациентов, у которых врач решит продолжить лечение бетагистином на протяжении более 60 дней, период последующего наблюдения проводиться не будет и для них завершением участия в программе будет окончание периода терапии в ходе наблюдения в рамках программы.

[5] Включает пол, возраст, расу (европеоидная, негроидная, монголоидная, другая), вес и рост.

[6] Частота сердечных сокращений, артериальное давление (систолическое и диастолическое).

[7] Для более подробной информации по Шкале оценки тяжести вестибулярного головокружения и клинического ответа (SVVSLCRE) см. раздел 6.3, Рисунок 1 и Таблица 1.

[8] 4-бальная шкала, где 4 =выраженное улучшение, 3 = значительное улучшение, 2 = незначительное улучшение и 1 = ухудшение.

[9] Через 30 дней после приема последней дозы препарата в ходе наблюдения в рамках программы/преждевременного завершения программы проводится телефонный контакт с пациентом для оценки возможных серьезных нежелательных явлениях. В случае выявления серьезных НЯ проводится сбор анамнеза и детальная оценка НЯ.

7.5.1 Поставки препарата

В рамках данной постмаркетинговой, наблюдательной программы, Спонсор не будет поставлять Бетасерк® в клинический центр. Препарат будет назначать врач, согласно утвержденной инструкции по применению препарата каждой из стран участников, в рамках рутинной клинической практики.

7.5.2 Процедуры наблюдательной программы

Визит 1, Сбор исходных данных / Начало терапии:

Будут проводиться следующие процедуры:

- Подписание и датирование согласия пациента на использование, и обработку персональных данных
- Оценка критериев включения/исключения

- Сбор демографических данных
- Регистрация даты начала лечения бетагистина дигидрохлоридом (Бетасерк®) (может отличаться от даты включения в исследование, пожалуйста, смотрите раздел 7.1.1)
- Регистрация сопутствующих препаратов
- Сбор анамнеза
- Присвоение идентификационного номера пациента
- Проведение физикального обследования, включая определение основных витальных функций (частота сердечных сокращений, систолическое и диастолическое артериальное давление)
- Диагноз по МКБ-10, симптомом которого является вестибулярное головокружение
- Регистрация числа эпизодов головокружения в течение прошедшего месяца
- Оценка клинического состояния пациента по шкале SVVSLCRE
- Мониторинг серьезных НЯ

Визит 2, до 30-го дня после приема первой дозы Бетасерк®:

Будут выполняться следующие процедуры:

- Регистрация сопутствующих препаратов
- Оценка клинического состояния пациента по шкале SVVSLCRE
- Регистрация числа эпизодов головокружения в течение прошедшего месяца
- Оценка общего клинического ответа и регресса связанных с головокружением симптомов по 4-бальной шкале
- Мониторинг серьезных НЯ

Визит 3, до 60-го дня после приема первой дозы Бетасерк®, завершение периода терапии в ходе наблюдения в рамках программы / преждевременное завершение участия в программе:

Будут выполняться следующие процедуры:

- Регистрация сопутствующих препаратов
- Оценка клинического состояния пациента по шкале SVVSLCRE
- Регистрация числа эпизодов головокружения в течение прошедшего месяца
- Оценка общего клинического ответа и регресса связанных с головокружением симптомов по 4-бальной шкале
- Мониторинг серьезных НЯ

Визит 4, 30 (±7) день после приема последней дозы Бетасерк®:

- Регистрация сопутствующих препаратов
- Регистрация числа эпизодов головокружения в течение прошедшего месяца
- Мониторинг серьезных НЯ

Визит 5, 60 (±7) день после приема последней дозы Бетасерк®:

- Регистрация сопутствующих препаратов
- Регистрация числа эпизодов головокружения в течение прошедшего месяца

8.0 Нежелательные явления

8.1 Определение нежелательного явления и категорий серьезного нежелательного явления

Нежелательное явление (НЯ) − любое неблагоприятное с медицинской точки зрения событие у пациента, которое необязательно связано с приемом исследуемого препарата. Нежелательное явление, следовательно, может представлять собой объективный неблагоприятный или непредсказуемый признак (включая отклонения в лабораторных показателях), субъективный симптом или заболевание, связанное по времени возникновения с назначением исследуемого препарата, независимо от того, имеется ли причинно-следственная связь между таким явлением и исследуемым препаратом.

Причинами НЯ могут быть применение лекарственного препарата (согласно информации, представленной в инструкции по медицинскому применению препарата), а также передозировка препарата, злоупотребление или отмена препарата. Любое ухудшение имеющегося состояния или заболевания считается нежелательным явлением.

Если нежелательное явление соответствует любому из следующих критериев, то оно считается **серьезным нежелательным явлением (СНЯ)**:

**Смерть субъекта:**	Явление, которое привело к смерти субъекта.
**Жизнеугрожающее состояние:**	Явление, которое, по мнению исследователя, без предпринятого медицинского вмешательства, привело бы к смерти. Это не относится к явлениям, которые могли бы быть привести к смерти, если бы наблюдались в более тяжелой форме.
**Госпитализация:**	Явление, которое стало причиной госпитализации любой продолжительности. К госпитализации не относятся посещения поликлиники или отделения скорой помощи.

**Продление госпитализации:**	Явление, которые развилось во время госпитализации субъекта и привело к увеличению продолжительности госпитализации.
**Врожденные пороки развития:**	Порок развития, выявленный во время или после рождения, или любой порок, который привел к самопроизвольному аборту.
**Продолжительная или значительная потеря трудоспособности/инвалидность**	Явление, которое приводит к состоянию, оказывающему существенное негативное влияние на повседневную деятельность субъекта. Понятие инвалидность не подразумевает включение таких относительно незначительных медицинских состояний и симптомов как головная боль, тошнота, простуда, случайная травма (например, растяжение связки).
**Важное медицинское явление, требующее терапевтического или хирургического вмешательства для предупреждения серьезного исхода:**	Важное с медицинской точки зрения явление, которое не представляют в данный момент угрозу для жизни и не является причиной смерти/госпитализации, но, по мнению врача, может представлять угрозу для субъекта или потребовать терапевтического или хирургического вмешательства для предотвращения описанных выше явлений (т.е. смерти субъекта, жизнеугрожающему состоянию, госпитализации, продлению госпитализации, врожденному пороку развития или продолжительному/существенному нарушению трудоспособности/инвалидности). Кроме того, любой плановый или самопроизвольный аборт или мертворождение считаются важными медицинскими явлениями. Примерами таких явлений являются аллергический бронхоспазм, требующий интенсивной терапии в отделении скорой помощи или дома, отклонения в показателях крови или судороги, не требующие госпитализации, или развитие зависимости или злоупотребление препаратом.

8.2 Степень тяжести

Врач будет использовать следующие определения для оценки степени тяжести всех нежелательных явлений, регистрируемых для оценки конечной точки, и всех серьезных нежелательных явлений.

**Легкая:**	Нежелательное явление является преходящим, легко переносится пациентом.
**Умеренная:**	Нежелательное явление вызывает дискомфорт у пациента и препятствует обычной повседневной деятельности.
**Высокая (тяжелые явления):**	Нежелательное явление в значительной степени препятствует обычной повседневной деятельности пациента, может приводить к инвалидности или угрожать жизни.

8.3 Связь с фармацевтическим препаратом

Исследователь будет использовать следующие определения для оценки причинно-следственной связи нежелательного явления с исследуемым препаратом:

**Вероятная связь**	При развитии нежелательного явления имеются данные, позволяющие предположить наличие причинно-следственной связи между исследуемым препаратом и нежелательным явлением.
**Отсутствие вероятной связи**	При развитии нежелательного явления отсутствуют данные, которые бы позволили предположить наличие причинно-следственной связи между исследуемым препаратом и нежелательным явлением.

Если исследователь указывает на отсутствие вероятной причинно-следственной связи с исследуемым препаратом, должно быть представлено альтернативное объяснение возникновения нежелательного явления.

8.4 Период регистрации серьезных нежелательных явлений

Необходимо извещать компанию «Эбботт» обо всех серьезных нежелательных явлениях, которые отмечались с момента подписания пациентом согласия на использование и обработку персональных данных и до 30-го дня после приема последней дозы исследуемого препарата.

8.5 Сообщения о серьезных нежелательных явлениях

В случае развития серьезного нежелательного явления, врач:

- для явлений, отмечавшихся у пациентов, принимавших препарат Эбботт, известит контактное лицо компании Эбботт (приведены ниже) в течение 24 часов, после того как о таком явлении стало известно.

Контактная информация представителей Эбботт:

Россия:

Эксперт по безопасности:

Алексей Ряхин,
Менеджер по фармаконадзору

Эбботт Лэбораториз
ТЦ "Метрополис"
Ленинградское шоссе, д. 16А, строение 1, Москва 125171 Российская Федерация
Тел.: +7 495 258 4280
Факс: +7 926 101 49 64
Факс: +7 495 258 4281
aleksey.riahin@abbott.com

Эксперт по безопасности (заместитель):

Антон Писарихин
Специалист по фармаконадзору

Эбботт Лэбораториз
ТЦ "Метрополис"
Ленинградское шоссе, д. 16А, строение 1, Москва 125171 Российская Федерация
Тел.: +7 495 258 4280
Моб.: +79261979966
Факс: +7 495 258 4281
anton.pisarihin@abbott.com

Украина:

Эксперт по безопасности

Александр Молодецкий,
Специалист по безопасности

Эбботт Украина,
ул. Жилянская, д. 110, 2й этаж
Киев, 01032 Украина

Тел: +380 44 498-60-80
Факс: +380 44 498-60-84

Эксперт по безопасности (заместитель):

Анна Иванова,
Специалист по безопасности

Эбботт Украина,
ул. Жилянская, д. 110, 2й этаж
Киев, 01032 Украина

Тел: +380 44 498-60-80
Факс: +380 44 498-60-84

8.6 Сообщение о беременности

Будет регистрироваться информация о беременностях среди субъектов наблюдательной программы, а также об исходах беременности.

Беременность у субъекта наблюдательной программы не считается нежелательным явлением. Однако любые осложнения беременности (например, плановый или самопроизвольный аборт, мертворождение, врожденные пороки развития и др.) считаются серьезными нежелательными явлениями, в таких случаях необходимо известить Эбботт о таких явлениях в течение 24 часов.

При установлении факта беременности у субъекта наблюдательной программы, врач должен известить об этом контактное лицо Эбботт (указанное в Разделе 8.5) в течение 24 часов, после того как врачу стало известно о случае беременности.

9.0 Этика и качество

Перед включением пациента в данную программу у него будет получено письменное согласие на использование и обработку персональных данных; пациенты не желающие предоставить такое письменное согласие в программу включаться не будут. При этом следует принимать все доступные меры,

направленные на сохранение конфиденциальности личной информации субъекта (например, имени, адреса и др.).

Россия: протокол и значимые документы программы будут предоставляться для рассмотрения и утверждения в уполномоченные государственные органы и центральный и/или местный независимый этический комитет (НЭК) (если применимо); включение пациентов в программу начнется только после получения письменного подтверждения НЭК.

Украина: Эбботт предоставит протокол и значимые документы наблюдательной программы для рассмотрения и утверждения в Государственный экспертный центр Министерства здравоохранения Украины. В обязанности каждого исследователя входит получение до начала наблюдательной программы заключения Независимого местного комитета по этике, если такое требуется.

Все данные будут регистрироваться таким образом, чтобы избежать раскрытия персональной информации об участниках программы, с целью сохранения конфиденциальности субъектов в течение всего периода проведения программы.

Врач-исследователь несет ответственность за контроль качества и надлежащую работу системы по контролю качества, чтобы убедиться, что все этапы данной программы проводятся в соответствии с протоколом, стандартами Надлежащей клинической практики и всеми применимыми местными законами и правилами.

Представитель Эбботт будет проверять индивидуальные регистрационные карты после их заполнения в системе ЭСД. Для уточнения несогласованных данных исследователю будет направлен запрос. Любые необходимые корректировки базы данных будут производиться по соответствующим процедурам системы ЭСД.

10.0 Индивидуальная регистрационная карта

В настоящей программе будут использоваться электронная Индивидуальная регистрационная карта (эИРК). эИРК будут заполняться для каждого субъекта, включенного в данную программу. эИРК будут использованы для сбора и передачи данных в Эбботт (или контрактным исследовательским организациям для обработки) и регуляторные органы, если требуется. Доступ к эИРК будет осуществляться через веб-сайт. Все данные эИРК должны вводиться корректно только уполномоченными лицами, прошедшими обучение. В эИРК автоматически сохраняются все внесенные изменения. Все несогласованные данные в эИРК будут направляться исследователю для уточнения в форме запроса. Вся информация, введенная в эИРК, должна также быть отражена в первичной медицинской документации субъекта.

Исследователь должен хранить данные субъекта в его первичной медицинской документации в соответствии с установленной практикой. Эта документация в данной программе будет использоваться в качестве исходной. Главный исследователь будет проверять эИРК на полноту и точность заполнения, а также подписывать карты каждого субъекта электронной подписью. Персонал Эбботт (или уполномоченные лица) также будут периодически проверять эИРК на полноту заполнения, корректность и применимость.

11.0 План анализа данных

Общие принципы

В настоящем разделе представлены основные аспекты планируемого статистического анализа данных, полученных в ходе программы. Так как настоящая программа является открытой, нерандомизированной, первичный анализ будет проводиться методами описательной статистики. Количественные показатели будут представлены количеством пациентов с расчетом среднего, стандартного отклонения, медианы, 1-го и 3-го квартилей, а также указанием минимальных и

максимальных значений. Качественные переменные будут представлены в виде абсолютного числа и доли пациентов в каждой категории. Кроме того, для расчетных значений будут определяться двусторонние 95% доверительные интервалы. В некоторых случаях для наглядного представления полученных данных могут быть построены графики.

Популяция анализа

Общая популяция пациентов будет включать всех пациентов, подписавших согласие на использование и обработку персональных данных. Эта популяция будет использоваться для представления распределения субъектов.

Полная анализируемая популяция пациентов будет включать пациентов, прошедших Визит 1 и получивших хотя бы одну дозу бетагистина дигидрохлорида (Бетасерк®). Эта популяция будет использоваться для анализа результатов программы.

Обзор демографических и исходных характеристик

Для демографических и исходных характеристик будут применяться методы описательной статистики (как указано выше) для общей популяции исследования и для каждой страны отдельно (Россия и Украина).

- Для возраста будут представлено количество пациентов с расчетом среднего, стандартного отклонения, медианы, 1-го и 3-го квартилей, а также указаны минимальные и максимальные значения. Будут рассчитаны доли пациентов старше, младше и равные медианному значению возраста.
- Для пола и рас будут указаны абсолютные числа и доли пациентов.
- Вес и рост будут использоваться для определения индекса массы тела. Для индекса массы тела будут представлены количество пациентов с

расчетом среднего, стандартного отклонения, медианы, 1-го и 3-го квартилей, а также указаны минимальные и максимальные значения.

- Для сопутствующих препаратов будут указаны абсолютные числа и доли пациентов, их получавшие.
- Для значимых данных анамнеза и физикального обследования также будут указаны абсолютные числа и доли пациентов.
- Для частоты сердечных сокращений, артериального давления (систолического и диастолического) будут представлены количество пациентов с расчетом среднего, стандартного отклонения, медианы, 1-го и 3-го квартилей, а также указаны минимальные и максимальные значения. Для пациентов с потенциально значимыми отклонениями показателей основных витальных функций организма будут указаны абсолютные числа и доли пациентов.

Ниже представлены критерии для определения потенциально значимых отклонений в показателях основных витальных функций:

Критерии для определения потенциально значимых отклонений в показателях основных витальных функций:

Показатель (единицы)		Диапазон	
Артериальное давление	Систолическое давление (мм рт. ст.)	80	160
	Диастолическое давление (мм рт. ст.)	50	100
Частота сердечных сокращений (уд/мин)		40	150

- Будут указаны абсолютные числа и доли пациентов с диагнозами по МКБ-10, симптомом которых является вестибулярное головокружение.

Анализ первичной конечной точки

1. Количество и доля субъектов с клиническим ответом в конце периода терапии в ходе наблюдения в рамках программы, при оценке по шкале SVVSLCRE. Будет представлен двухсторонний 95% доверительный интервал для пропорции.

Вторичные конечные точки

Анализ вторичных конечных точек будет выполнен только для получения дополнительных данных, и не предполагает доказательство преимуществ препарата для достижения данных конечных точек. Следовательно, величины p и доверительные интервалы для вторичных конечных точек будут представлены исключительно в описательных целях.

1. Количество и доля пациентов в каждой категории по шкале SVVSLCRE при включении в наблюдательную программу и на момент завершения терапии в ходе наблюдения в рамках программы будут представлены среди пациентов, получавших лечение бетагистина дигидрохлоридом в течение ≤30 дней, и среди пациентов, получавших бетагистина дигидрохлорид >30 дней, но ≤60 дней.

2. Изменение в степени тяжести вестибулярного головокружения по шкале SVVSLCRE на момент завершения терапии в ходе наблюдения в рамках программы по сравнению с визитом включения в программу будет представлено с указанием медианы, 1-го и 3-го квартилей. Сравнение между исходными показателями и значениями при завершения терапии в ходе наблюдения в рамках программы будут производить с помощью Т-Критерия Уилкоксона для зависимых переменных. Для каждого сравнения будут представлены значения критерия и величины p. Результаты анализа с величиной p равной или менее 0,05 будут представляться как «статистически значимые».

3. Изменение степени тяжести вестибулярного головокружения по шкале SVVSLCRE на Визите 2 по сравнению с визитом включения в программу будет представлено с указанием медианы, 1-го и 3-го квартилей. Сравнение между исходными показателями и значениями на Визите 2 будут выполнять с помощью Т-Критерия Уилкоксона для зависимых переменных. Для каждого сравнения будут представлены значения критерия и величины p. Результаты анализа с величиной p равной или менее 0,05 будут представляться как «статистически значимые».

4. Изменение частоты эпизодов вестибулярного головокружения (выражается в количестве приступов в месяц) на момент завершения терапии в ходе наблюдения в рамках программы по сравнению с визитом включения в программу будет приводиться с указанием количества пациентов, среднего, стандартного отклонения, медианы, 1-го и 3-го квартилей, а также минимальных и максимальных значений. Также будут представлены описательные статистики каждого показателя при включении в программу и на момент завершения терапии в ходе наблюдения в рамках программы. Сравнение между исходными показателями и значениями на момент завершения терапии в ходе наблюдения в рамках программы будут производиться с помощью двухстороннего парного t-критерия Стьюдента для зависимых переменных (если будет наблюдаться близкое к нормальному распределение данных) или с помощью Т-Критерия Уилкоксона для зависимых переменных (непараметрический критерий). Для каждого сравнения будут представлены значения критерия и величины p. Результаты анализа с величиной p равной или менее 0,05 будут представляться как «статистически значимые».

5. Изменение частоты эпизодов вестибулярного головокружения (выражается в количестве приступов в месяц) на Визите 2 по сравнению с визитом включения в программу будет приводиться с указанием количества пациентов, среднего, стандартного отклонения, медианы, 1-го и 3-го квартилей, а также минимальных и максимальных значений. Также будут представлены описательные статистики

каждого критерия при включении в программу и на Визите 2. Сравнение между исходными показателями и значениями на Визите 2 будут производиться с помощью двухстороннего парного t-критерия Стьюдента для зависимых переменных (если будет наблюдаться близкое к нормальному распределение данных) или с помощью Т-Критерия Уилкоксона для зависимых переменных (непараметрический критерий). Для каждого сравнения будут представлены значения критерия и величины р. Результаты анализа с величиной р равной или менее 0,05 будут представляться как «статистически значимые».

6. Общий клинический ответ при оценке врачом по четырехбальной шкале будет представлен с указанием количества и доли пациентов в каждой категории на момент завершения терапии в ходе наблюдения в рамках программы среди пациентов, получавших лечение бетагистина дигидрохлоридом в течение ≤30 дней, и на Визите 2 и 3 среди пациентов, получавших бетагистина дигидрохлорид >30 дней, но ≤60 дней.

7. Общий клинический ответ при оценке пациентом по четырехбальной шкале будет представлен с указанием количества и доли пациентов в каждой категории на момент завершения терапии в ходе наблюдения в рамках программы среди пациентов, получавших лечение бетагистина дигидрохлоридом в течение ≤30 дней, и на Визите 2 и 3 среди пациентов, получавших бетагистина дигидрохлорид >30 дней, но ≤60 дней.

8. Клинический ответ, определяемый как регресс связанных с головокружением симптомов (звон в ушах, нарушение слуха, тошнота, рвота, слабость и головная боль), при оценке врачом по четырехбальной шкале будет представлен с указанием количества и доли пациентов в каждой категории на момент завершения терапии в ходе наблюдения в рамках программы среди пациентов, получавших лечение

бетагистина дигидрохлоридом в течение ≤30 дней, и на Визите 2 и 3 среди пациентов, получавших бетагистина дигидрохлорид >30 дней, но ≤60 дней.

9. Клинический ответ, определяемый как регресс связанных с головокружением симптомов (звон в ушах, нарушение слуха, тошнота, рвота, слабость и головная боль), при оценке пациентом по четырехбальной шкале будет представлен с указанием количества и доли пациентов в каждой категории на момент завершения терапии в ходе наблюдения в рамках программы среди пациентов, получавших лечение бетагистина дигидрохлоридом в течение ≤30 дней, и на Визите 2 и 3 среди пациентов, получавших бетагистина дигидрохлорид >30 дней, но ≤60 дней.

10. Изменение частоты эпизодов вестибулярного головокружения (в виде количества приступов в месяц) в конце первого месяца периода последующего наблюдения по сравнению с завершением периода терапии в ходе наблюдения в рамках программы, а также в конце второго месяца периода последующего наблюдения по сравнению с завершением периода терапии в ходе наблюдения в рамках программы будут представлены для пациентов, получавших лечение бетагистина дигидрохлоридом в течение ≤30 дней, и для пациентов, получавших бетагистина дигидрохлорид>30 дней, но ≤60 дней. Будут представлены количество пациентов, среднее, стандартное отклонение, медиана, 1-й и 3-й квартиль, а также минимальные и максимальные значения. Также будут представлены описательные статистики каждого показателя на момент завершения терапии в ходе наблюдения в рамках программы и через 1 и 2 месяца периода последующего наблюдения. Сравнения между показателями на момент завершения терапии в ходе наблюдения в рамках программы и через 1 и 2 месяца периода последующего наблюдения будут производить с помощью двухстороннего парного t-критерия Стьюдента для зависимых переменных (если будет наблюдаться близкое к нормальному распределение данных) или с помощью Т-Критерия Уилкоксона для зависимых переменных (непараметрический критерий). Для каждого сравнения будут

представлены значения критерия и величины p. Результаты анализа с величиной p равной или менее 0,05 будут представляться как «статистически значимые».

11. Будут указаны абсолютные числа и доли пациентов с диагнозами по МКБ-10, симптомом которых является вестибулярное головокружение.

Другие значимые аспекты планируемого анализа данных

Анализ данных будет включать дополнительные поисковые цели, для которых подробное описание статистических методов будет представлено в отдельном документе − Плане статистического анализа. Планируется изучение следующих поисковых целей:

1. Оценка эффектов препарата Бетасерк® и течение вестибулярного головокружения в периоде последующего наблюдения в подгруппах пациентов по основному диагнозу согласно МКБ-10.

2. Характеристика сопутствующих препаратов для лечения вестибулярного головокружения, применяемых на протяжении данной программы в подгруппах пациентов по основному диагнозу согласно МКБ-10.

3. Оценка эффектов Бетасерк® и течения вестибулярного головокружения в период последующего наблюдения в подгруппах пациентов по половой принадлежности и по странам (Россия и Украина).

Кроме того, количество и доля пациентов с серьезными нежелательными явлениями (включая случаи смерти), а также с нежелательными явлениями, которые приводили к отмене исследуемого препарата, будут представлены в виде таблиц с разделением по классу систем органов MedDRA и предпочтительным терминам MedDRA.

Объем выборки

Основываясь на опубликованных данных исследования Gananca et al. в Acta Oto-Laryngologica, 2009 [17], доля пациентов с болезнью Меньера, у которых достигался контроль головокружения (клинический уровень 4 и 3) через 12 недель лечения таблетками бетагистина дигидрохлорида, составляет 88%. Benecke at al. [18] установили, что общая эффективность бетагистина в клинической практике при продолжительности терапии 3 месяца являлась отличной у 36,6% из 1753 пациентов (n = 641) и хорошей у 49,6% пациентов (n = 870), всего 86,2%. По мнению исследователя эффективность лечения была отличной у 38,6% пациентов (n = 675 из 1747) и хорошей у 50,5% пациентов (n = 883), всего 89,1%.

При разработке настоящей наблюдательной программы, предполагали, что не менее чем у 80% общей выборки субъектов будет достигнут клинический ответ через 60 дней лечения таблетками бетагистина дигидрохлорида. При размере выборки 246 субъектов (без учета досрочно выбывших пациентов), нормальная аппроксимация двухстороннего 95% доверительного интервала для одной пропорции при нормальном распределении будет составлять 5,0% (т.е. половина ширины 95% доверительного интервала) и будет в пределах 80% [19; 20]. При 20% досрочно выбывших субъектов, общая исследуемая популяция (пациенты, получившие хотя бы одну дозу бетагистина дигидрохлорида (Бетасерк®) и для которых имеются данные хотя бы одного обследования после включения в программу) составит 310 пациентов.

Таким образом, оптимальный объем выборки для данной наблюдательной программы составит 310 пациентов.

12.0 Итоговый отчет и публикации

По окончании данной программы, в сотрудничестве с главным исследователем будет составлен отчет (Отчет по наблюдательной программе будет представлен в течение 10 месяцев после последнего визита последнего пациента). Данный отчет будет содержать описание целей наблюдательной программы, методологии, а также полученные результаты и выводы. Заполненные индивидуальные регистрационные карты и отчет по наблюдательной программе являются конфиденциальной собственностью Эбботт Лэбораториз, их содержание не может разглашаться третьем лицам в любой форме (включая публикации и презентации) без предварительного письменного разрешения Эбботт Лэбораториз. Результаты данного постмаркетинговой программы могут публиковаться Эбботт Лэбораториз или исследователями только после получения письменного согласия от Эбботт Лэбораториз.

13.0 Список литературы

1. Морозова С.В., Зайцева О.В., Налетова Н.А. Головокружение как медико-социальная проблема. // РМЖ. - 2002. - № 4. – с.164.
2. Oosterland W.J. Betahistine dihydrochloride in the treatment of vertigo of peripheral vestibular origin. A double-blind placebocontrolled study. J Laryngol Otol 1984; 98:37–41.
3. Yardley L, Owen N, Nazareth I, Luxon L. Prevalence and presentation of dizziness in a general practice community sample of working age people. Br J Gen Pract. 1998; 48(429):1131–1135.
4. Colledge N.R., Barr–Hamilton R.M. Evaluation of investigations to diagnose the cause of dizziness in elderly people: a community based controlled study. BMJ. 1996; 313:788 – 92.
5. Claussen C.F. Development of Clinical Neurootological Network Diagnostics During the Last Three Decades. Int Tinnitus J. 2000; 6(2):77.
6. Neuhauser HK Epidemiology of vestibular vertigo. Neurology 2005; 65:898-904.
7. Zamergrad M.V .Vestibular vertigo Neurology, neuropsychiatry, psychosomatics, № 1, 2009, 14-18.
8. Алексеева Н.С. // Материалы симпозиума “Головокружение: современные подходы к решению проблемы” 8–го съезда неврологов России. – М. – 2001. – с. 2 – 5.
9. Благовещенская Н.С. Отоневрологические симптомы и синдромы. – М.: Медицина. – 1990. – 432 с.
10. Thompson T L, Amedee R “Vertigo: A review of common peripheral and central vestibular disorders” The Ochsner Journal 2009, 9: 20-26.
11. Brandt T. Vertigo. Its Multicensory Syndromes. London: Springer, 2000, p. 503.

12. Brandt T, Dieterich M. Vertigo and Dizziness: common complains. London: Springer, 2004; p 208.

13. Debasish Burman1, Saileswar Goswami, Pallab Kumar Majumdar; A Study on Peripheral Vertigo in a Kolkata Based Hospital. Indian Journal of Otolaryngology and Head and Neck Surgery Vol. 54 No. 2, April - June 2002.

14. Alexander G. Reeves, M.D. Disorders of the Nervous System, 2008 Chapter 14.

15. Lacour M, Sterkers O. Histamine and betahistine in the treatment of vertigo. Elucidation of mechanisms of action. CNS Drugs 2001; 15: 853-70.

16. Strupp M, Hupert D, Frenzel C, Wagner J, Hahn A, Jahn K, Zingler VC, Mansmann U, Brandt T. Long-term prophylactic treatment of attacks of vertigo in Menière's disease--comparison of a high with a low dosage of betahistine in an open trial. Acta Otolaryngol. 2008; 128(5):520-4.

17. Ganança MM, Caovilla HH, Ganança FF. Comparable efficacy and tolerability between twice daily and three times daily betahistine for Meniere’s disease. Acta Otolaryngol. 2009; 129(5):487-92.

18. Benecke H. et al. Effects of Betahistine on Patient-Reported Outcomes in Routine Practice in Patients with Vestibular Vertigo and Appraisal of Tolerability: Experience in the OSVaLD Study. International Tinnitus Journal. 2010; 16(1):14-24.

19. Fleiss, J. L., Levin, B., Paik, M.C. 2003. Statistical Methods for Rates and Proportions. Third Edition. John Wiley & Sons. New York.

20. Newcombe, R. G. 1998. 'Two-Sided Confidence Intervals for the Single Proportion: Comparison of Seven Methods.' Statistics in Medicine, 17, pp. 857-872.

21. Neuhauser HK, von Brevern M, Radtke A, Lezius F, Feldmann M, Ziese T, Lempert T. Epidemiology of vestibular vertigo: a neurotologic survey of the general population. Neurology. 2005; 65(6):898-904.

22. Sloane P.D. Dizziness in primary care: results from the National Ambulatory Medical Care Survey. J Fam Pract 1989; 29:33-38.

23. Redon C, Lopez C, Bernard-Demanze L, Dumitrescu M, Magnan J, Lacour M, Borel L. Betahistine treatment improves the recovery of static symptoms in patients with unilateral vestibular loss. J Clin Pharmacol. 2011; 51(4):538-48.

24. Lacour M, Sterkers O. Histamine and betahistine in the treatment of vertigo: elucidation of mechanisms of action. CNS Drugs. 2001; 15(11):853-70.

14.0 Приложение: Поправка к протоколу 1: Перечень изменений

<u>Изменения</u> Поправка 1, версия от 10 декабря 2012.

1.1 Поправка к протоколу 1: Обзор изменений

Данный раздел включен для описания изменений, содержащихся в Поправке 1.

Раздел 7.5 Проведение наблюдательной программы

Изображение 1: Общий план проведения наблюдательной программы ранее читалось:

Изображение 2: Общий план проведения наблюдательной программы

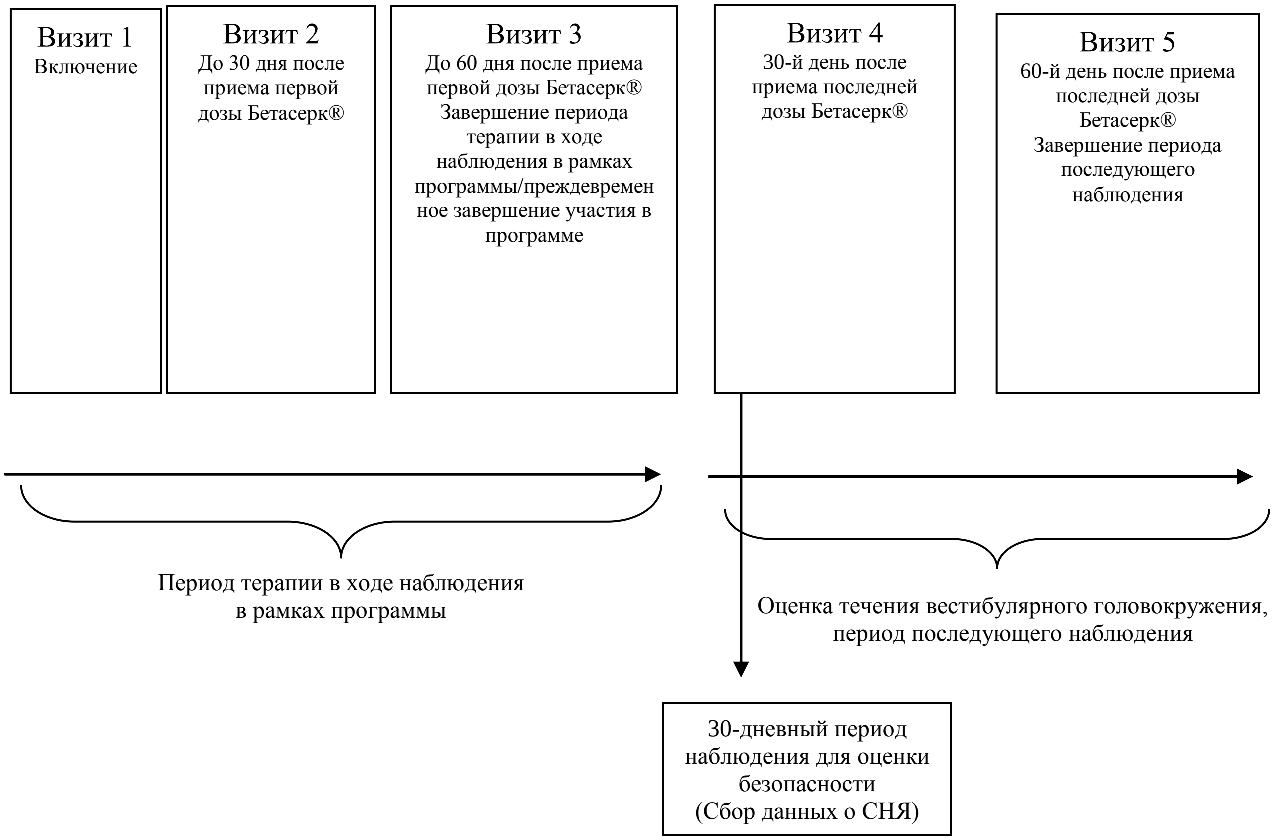

После изменения следует читать:

Рисунок 2: Общий план проведения наблюдательной программы

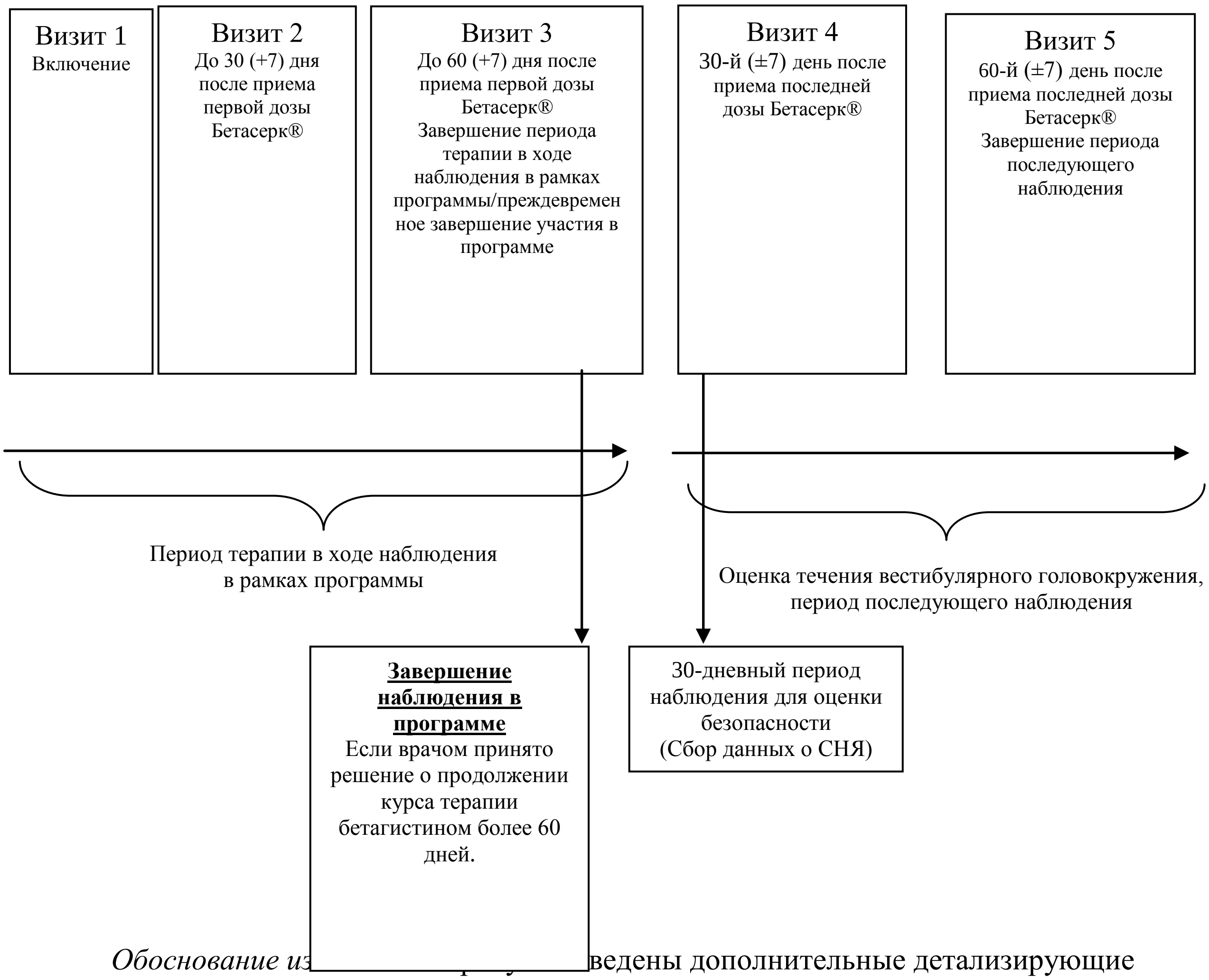

Обоснование из[illegible]ведены дополнительные детализирующие описания, поясняющие текстовую часть протокола; номер рисунка изменен на правильный, в соответствии с порядком появления рисунка в протоколе.

Таблица 1 Процедуры наблюдательной программы

		Период терапии в ходе наблюдения в рамках программы[1]		Оценка течения вестибулярного головокружения в период последующего наблюдения	
	Визит 1	Визит 2	Визит 3	Визит 4	Визит 5
		В течение 30 дней после приема первой дозы Бетасерк®[2]	В течение 60 дней после приема первой дозы Бетасерк®[2]	30й (±7) день после приема последней дозы Бетасерк®	60й (±7) день после приема последней дозы Бетасерк®
	Скрининг/ Включение, сбор исходных данных		Завершение периода терапии в рамках наблюдения в ходе программы [3,4]/ преждевременное завершение участия в программе		Завершение периода последующего наблюдения
Согласие на использование и обработку персональных данных	X				
Оценка критериев включения / исключения	X				
Регистрация даты начала применения бетагистина дигидрохлорида (Бетасерк®)	X				
Сбор демографических данных[5]	X				
Сбор анамнеза	X				
Регистрация сопутствующих препаратов	X	X	X	X	X
Физикальное обследование (включая оценку основных витальных функций организма[6])	X				
Диагноз по МКБ-10, симптомом которого является вестибулярное головокружение	X				
Количество приступов головокружения в месяц	X	X	X	X	X
Шкала оценки тяжести вестибулярного головокружения и клинического ответа (SVVSLCRE)[7]	X	X	X		
Общий клинический ответ и регресс связанных с вестибулярным головокружением симптомов по 4-бальной шкале[8]		X	X		
Мониторинг серьезных НЯ[9]	X	X	X	X	

После изменения следует читать:

Таблица 2 Процедуры наблюдательной программы

		**Период терапии в ходе наблюдения в рамках программы**[1]		**Оценка течения вестибулярного головокружения в период последующего наблюдения**	
	**Визит 1**	**Визит 2**	**Визит 3**	**Визит 4**	**Визит 5**
		В течение 30 дней после приема первой дозы Бетасерк®[2]	В течение 60 дней после приема первой дозы Бетасерк®[2]	30й (±7) день после приема последней дозы Бетасерк®	60й (±7) день после приема последней дозы Бетасерк®
	Скрининг/ Включение, сбор исходных данных		Завершение периода терапии в рамках наблюдения в ходе программы [3,4]/ преждевременное завершение участия в программе		Завершение периода последующего наблюдения
Согласие на использование и обработку персональных данных	X				
Оценка критериев включения / исключения	X				
Регистрация даты начала применения бетагистина дигидрохлорида (Бетасерк®)	X				
Сбор демографических данных[5]	X				
Сбор анамнеза	X				
Регистрация сопутствующих препаратов	X	X	X	X	X
Физикальное обследование (включая оценку основных витальных функций организма[6])	X				
Диагноз по МКБ-10, симптомом которого является вестибулярное головокружение	X				
Количество приступов головокружения в месяц	X	X	X	X	X
Шкала оценки тяжести вестибулярного головокружения и клинического ответа (SVVSLCRE)[7]	X	X	X		
Общий клинический ответ и регресс связанных с вестибулярным головокружением симптомов по 4-бальной шкале[8]		X	X		
Мониторинг серьезных НЯ[9]	X	X	X	X	

Обоснование изменений: номер таблицы изменен на правильный, в соответствии с порядком изложения таблицы в протоколе.

Раздел 7.5.2 Процедуры наблюдательной программы

Содержание Визита 4 и Визита 5 ранее читалось:

Визит 4, 30 (±7) день после приема последней дозы Бетасерк®:

- Регистрация сопутствующих препаратов
- Регистрация числа эпизодов головокружения в течение прошедшего месяца
- Оценка общего клинического состояния пациента по 4-бальной шкале
- Мониторинг серьезных НЯ

Визит 5, 60 (±7) день после приема последней дозы Бетасерк®:

- Регистрация сопутствующих препаратов
- Регистрация числа эпизодов головокружения в течение прошедшего месяца
- Оценка общего клинического состояния пациента по 4-бальной шкале

После изменения следует читать:

Визит 4, 30 (±7) день после приема последней дозы Бетасерк®:

- Регистрация сопутствующих препаратов
- Регистрация числа эпизодов головокружения в течение прошедшего месяца
- Мониторинг серьезных НЯ

Визит 5, 60 (±7) день после приема последней дозы Бетасерк®:

- Регистрация сопутствующих препаратов
- Регистрация числа эпизодов головокружения в течение прошедшего месяца

Обоснование изменений: Из описания визитов 4 и 5 исключен пункт «Оценка общего клинического состояния пациента по 4-бальной шкале» в соответствии с перечнем процедур для данных визитов, предусмотренных в Таблице 2 Процедуры наблюдательной программы.

Раздел 9.0 Этика и качество ранее читался:

Украина: Эбботт предоставит протокол и значимые документы наблюдательной программы для рассмотрения и утверждения в Комитет по этике Министерства здравоохранения Украины. В обязанности каждого исследователя входит получение до начала наблюдательной программы заключения Независимого местного комитета по этике, если такое требуется.

После изменения следует читать:

Украина: Эбботт предоставит протокол и значимые документы наблюдательной программы для рассмотрения и утверждения в Государственный экспертный центр Министерства здравоохранения Украины. В обязанности каждого исследователя входит получение до начала наблюдательной программы заключения Независимого местного комитета по этике, если такое требуется.

Обоснование изменений: изменения в локальном законодательстве.

Эбботт Лэбораториз

Постмаркетинговая наблюдательная программа Протокол (P13-972)

Поправка 1

Постмаркетинговая наблюдательная программа по оценке эффектов препарата Бетасерк® (бетагистина дигидрохлорид) у пациентов с вестибулярным головокружением в рутинной клинической практике (VIRTUOSO)

Утверждено:

Автор протокола - Александр Владыкин/Старший специалист по клиническим исследованиям	Дата
Врач-координатор программы - Марина Матросова/Медицинский советник	Дата
Дополнительный рецензент - Руслан Нафеев/Медицинский советник	Дата
Статистик - Джос Нута/ Статистик	Дата
Директор проекта - Катинка Гейземан / Медицинский директор	Дата
